# Structural Basis and Mechanism of a Unique Haemophore in the Haem‐Iron Acquisition by *Riemerella anatipestifer*

**DOI:** 10.1002/advs.202412202

**Published:** 2025-01-30

**Authors:** Mengying Wang, Dandan Zhang, Xiu Tian, Jiangyang Tong, Yizhou Yao, Mingshu Wang, Dekang Zhu, Renyong Jia, Shun Chen, Xinxin Zhao, Shaqiu Zhang, Juan Huang, Xumin Ou, Bin Tian, Di Sun, Yu He, Zhen Wu, Songying Ouyang, Mafeng Liu, Anchun Cheng

**Affiliations:** ^1^ Engineering Research Center of Southwest Animal Disease Prevention and Control Technology Ministry of Education of the People's Republic of China Chengdu 611130 China; ^2^ Key Laboratory of Animal Disease and Human Health of Sichuan Province Chengdu 611130 China; ^3^ International Joint Research Center for Animal Disease Prevention and Control of Sichuan Province Chengdu 611130 China; ^4^ Research Center of Avian Disease College of Veterinary Medicine Sichuan Agricultural University Chengdu 611130 China; ^5^ Key Laboratory of Microbial Pathogenesis and Interventions of Fujian Province University the Key Laboratory of Innate Immune Biology of Fujian Province Biomedical Research Center of South China Key Laboratory of OptoElectronic Science and Technology for Medicine of the Ministry of Education College of Life Sciences Fujian Normal University Fuzhou 350117 China

**Keywords:** haem, haemophore, iron, Riemerella anatipestifer

## Abstract

Several bacterial pathogens employ haemophores to scavenge haem from host haemoprotein to obtain an iron source. However, no homologues of well‐characterized haemophores are found in *Riemerella anatipestifer*, a bacterium belonging to the order *Flavobacteriales* that encodes haem uptake systems. Herein, a unique haemophore RhuH is characterized in this bacterium. *R. anatipestifer* used RhuH to grow when duck hemoglobin serves as the sole iron resource. RhuH is secreted as a component of outer membrane vesicles. Recombinant RhuH exhibited a high binding affinity for haem (*K*_d_ of 3.44 × 10^−11^ m) and can extract haem from duck hemoglobin. X‐ray crystallography elucidated the 3D structure of RhuH at 2.85 Å resolution, showing a dimeric conformation with each monomer exhibiting a unique structure. Structure modeling of RhuH‐haem, coupled with mutagenesis, haemin utilization, and binding affinity assays, show that haem is captured in the β‐barrel–like region, displaying the classic iron coordination. The RhuH homologues are predominantly distributed in *Weeksellaceae* and *Flavobacteriaceae*. Finally, the homologues of RhuH in *Riemerella columbina*, *Flavobacterium columnare*, and *Flavobacterium soli* are used as a proof of concept, demonstrating that these homologues exhibit conserved structures and functions.

## Graphical abstract

This study reveals the structural basis and mechanism of RhuH as a haemophore in *Riemerella anatipestifer*. Secreted by outer membrane vesicles (OMVs), RhuH captures haem with high affinity from host hemoglobin in two forms: a) released from OMVs, binding and transferring haem to outer membrane receptor, and b) haem‐bound on the OMVs surface, returning to the cell via membrane fusion.

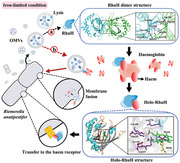

## 1 Introduction

Iron is an essential nutrient for the survival, proliferation, and virulence of most bacteria because of its role in respiration, DNA synthesis, and oxidoreduction reactions.^[^ ^1^ ^]^ In higher organisms, iron is sequestered in iron‐carrier proteins, transferrin and lactoferrin and in haem as haemoproteins, such as hemoglobin and haemopexin.^[^ ^2^ ^]^ In vertebrates, the haem in hemoglobin is the richest iron reservoir, and ≈70% of iron is stored in this protein.^[^ ^3^ ^]^ To fulfill their iron requirements, bacteria have developed multiple iron‐acquisition systems, including the FeoAB system,^[^ ^4^ ^]^ siderophore‐mediated iron‐utilization system^[^ ^5^ ^]^ and haem transport system.^[^ ^3b^ ^]^

By analogy with siderophores, bacteria secrete haemophores to scavenge exogenous haem from host haemoproteins. Haemophores are proteins exposed at the cell surface or secreted to transport haem from the host haemprotein to its specific receptor.^[^ ^6^ ^]^ Subsequently, the haem is transferred into the periplasm by TonB‐dependent receptor that requires the energy provided by TonB‐ExbB‐ExbD complex in the inner membrane. Subsequently, haem is transported into the cytoplasm through the inner membrane ABC transporter and is degraded by haem oxygenases, releasing iron for bacterial utilization.^[^ ^7^ ^]^ At least five types of haemophores have been characterized in Gram‐negative bacteria, including HasA (for haem acquisition system) of *Serratia marcescens*,^[^ ^8^ ^]^ HxuA (haem: haemopexin binding protein) of *Haemophilus influenzae*,^[^ ^9^ ^]^ HmuY and HusA (haem uptake system protein A) of *Porphyromonas gingivalis*,^[^ ^10^ ^]^ and HphA (hemophilin) of *Acinetobacter baumannii*.^[^ ^11^ ^]^ The first identified haemophore HasA of *S. marcescens* is a 19‐kDa protein that is secreted by a type I secretion pathway and can capture the haem from hemoglobin with high affinity (*K*_d_ = 1.8 × 10^−11^ m) and deliver this to the TonB‐dependent haem receptor HasR.^[^ ^12^ ^]^ HxuA of *H. influenzae* is localized on the outer membrane surface with a molecular weight of 100 kDa and is secreted by a two‐partner secretion machine.^[^ ^13^ ^]^ HxuA is strictly required for haem utilization from haemopexin, a serum glycoprotein with high haem affinity.^[^ ^14^ ^]^ Unlike HasA, HxuA does not bind to haem, but haem is released from haem–haemopexin upon the interaction of HxuA with haem–haemopexin, and the released haem is then captured by the TonB‐dependent receptor HxuC.^[^ ^13a^ ^]^ HusA of *P. gingivalis* has a molecular weight of 21 kDa and is localized in the outer membrane and outer membrane vesicles (OMVs).^[^ ^10^, ^15^ ^]^ HusA utilizes haem from hemoglobin and can also bind various abiotic and metal‐free porphyrins to mediate the uptake of essential porphyrins.^[^ ^10^, ^16^ ^]^ HmuY of *P. gingivalis* is initially anchored to the bacterial cell surface and subsequently released as a soluble protein following limited proteolytic cleavage.^[^ ^15^, ^17^ ^]^ HmuY sequesters haem from host haemoproteins or haem binding proteins produced by cohabiting bacteria with a *K*_d_ <10^−9^ m.^[^ ^18^ ^]^ Subsequently, the haem is delivered to the TonB‐dependent outer membrane receptor HmuR (haemin utilization receptor), facilitating its transport into the cell.^[^ ^10b^ ^]^ HphA of *A. baumannii* is secreted by the type XI secretion system Slam and can extract haem from both hemoglobin and serum albumin.^[^ ^11^ ^]^ HphA then facilitates the internalization of haem into the bacterial cell through an interaction with its specific TonB‐dependent receptor, HphR (hemophilin receptor).^[^ ^11^ ^]^ These five types of haemophores do not show significant sequence or structural similarities and have their own unique mechanisms for sequestering haem from host haem binding proteins.

*R. anatipestifer*, a Gram‐negative bacterium belonging to the family *Weeksellaceae* within the order *Flavobacteriales*, mainly infects domestic ducks, geese, turkeys, and chickens.^[^ ^19^ ^]^ *R. anatipestifer* requires haem for growth and encodes haem utilization systems.^[^ ^20^ ^]^ In our previous study, *R. anatipestifer* CH‐1 was shown to encode the outer membrane protein RhuA (*R. anatipestifer* haemin uptake protein A), which facilitates haem transport by a TonB2‐dependent haem receptor RhuR (*R. anatipestifer* haemin uptake receptor). However, the recombinant RhuA protein cannot remove haem from duck hemoglobin directly.^[^ ^20a^ ^]^ Consequently, we hypothesized that other factors, such as proteases or proteins, from *R. anatipestifer* mediate the release of haem from duck hemoglobin.

In this study, we characterized a unique haemophore in *Flavobacteriales* pathogen *R. anatipestifer*, referred to here as RhuH (*R. anatipestifer* haem uptake protein haemophore). RhuH is secreted as a component of the OMVs and functions as a novel haemophore that extracts haem from host hemoglobin. The structure of RhuH reveals a distinctive protein folding that is markedly different from that of other haemophores, and its haem binding mechanism utilizes haem iron coordination with high affinity. It is noteworthy that homologues of RhuH also was identified in other Flavobacteriales bacteria, including *Riemerella columbina*, *Flavobacterium columnare*, and *Flavobacterium soli*, exhibiting structural and functional similarities to RhuH. The RhuH of *R. anatipestifer* represents a novel haemophore in Gram‐negative bacteria that is essential for haem uptake.

## 2 Results

### 2.1 RhuH is Required for the Growth of *R. anatipestifer* CH‐1 Under Iron‐Limited Conditions Supplemented by Duck Hemoglobin and Haemin

**Figure 1:**
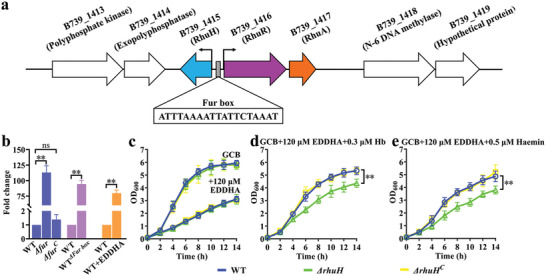
*R. anatipestifer* requires RhuH for growth in iron‐limited GCB medium supplemented by duck hemoglobin (Hb) and haemin. a) The organization of the locus B739_1415(RhuH)‐B739_1416(RhuR)‐B739_1417(RhuA) in the *R. anatipestifer* CH‐1 genome. The arrow represents the transcription direction of the genes. b) Relative mRNA levels of *rhuH* in *R. anatipestifer* CH‐1 (WT), *Δfur*, and *Δfur^C^* strains grown to the exponential phase, relative mRNA levels of *rhuH* in the WT and WT*^ΔFur box^* strains grown to the exponential phase, and relative mRNA levels of *rhuH* in *R. anatipestifer* CH‐1 in GCB and GCB with 120 µM EDDHA. c–e) Growth curves of WT (blue circles), *ΔrhuH* (green triangles), and *ΔrhuH^C^* (yellow square) strains under GCB (c), GCB containing 120 µm EDDHA (c), iron‐limited GCB supplemented with 0.3 µm duck hemoglobin (Hb) (d) and iron‐limited GCB supplemented with 0.5 µm haemin (e). Data represents mean plus standard deviations from three independent experiments each in biological triplicate. Statistical analyses were conducted using unpaired *t*‐test (b–e). ^**^, *p *< 0.01, ns: not significant.

We previously showed that the *rhuA‐rhuR* locus was involved in haem utilization from duck hemoglobin.^[^ ^20a^ ^]^ *rhuA* encodes an outer membrane haem binding protein that cannot extract haem from duck hemoglobin, while *rhuR* encodes a TonB2‐dependent haem receptor.^[^ ^20a^ ^]^ Interestingly, the upstream gene of *rhuR‐rhuA*, *B739_1415*, also exhibited upregulated expression in iron‐limited conditions according to RNA‐seq data.^[^ ^21^ ^]^ Sequence analysis showed that *B739_1415* and *rhuR‐rhuA* share a bidirectional promoter region, which is regulated by iron and contains a Fur box^[^ ^22^ ^]^ (**Figure** **1**a). The length of the promoter region is 122 bp, with the Fur box located 25 bp upstream of *B739_1415* and 78 bp upstream of *rhuR‐rhuA*. Compared with wild type (WT), the transcription of *B739_1415* was highly upregulated in the *Δfur* strain, the Fur box mutant strain (WT*^ΔFur box^*), and in iron‐limited conditions (Figure 1b), suggesting that *B739_1415* of *R. anatipestifer* CH‐1 was regulated by Fur and iron.

We then investigated whether B739_1415 is involved in haem utilization from duck hemoglobin. The *B739_1415* mutant had no effect on the growth of RA CH‐1 in GCB or GCB containing 120 µm of the iron chelator ethylenediamine‐*N,N′*‐bis[(2‐hydroxyphenyl)acetic acid] (EDDHA) (Figure 1c). However, compared with the WT strain, the *B739_1415* mutant exhibited defective growth in the iron‐limited medium supplemented with 0.3 µm duck hemoglobin (Hb) or 0.5 µm haemin (Figure 1d,e). Reintroduction of *B739_1415* under its native promoter into the *R. anatipestifer* CH‐1*ΔB739_1415* (*ΔrhuH*) mutant restored normal growth with duck hemoglobin or haemin as the sole source of iron (Figure 1d,e), confirming that *B739_1415* mutant was the main reason for the defective phenotype of duck hemoglobin or haemin utilization. On the basis of these observations and the following data, we renamed B739_1415 as RhuH (*R. anatipestifer* haem uptake protein haemophore).

### 2.2 RhuH is Secreted as a Protein in OMVs

**Figure 2:**
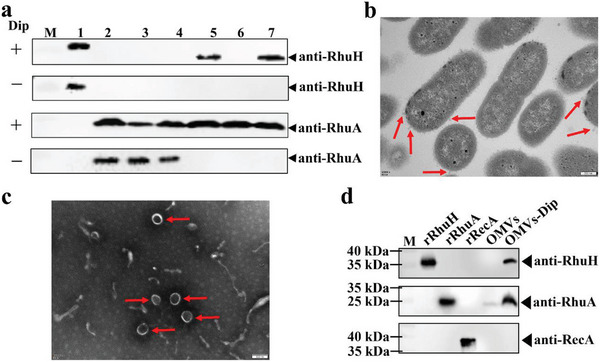
Subcellular distribution of RhuH in *R. anatipestifer* CH‐1. a) Bacterial cells were harvested from GCB media supplemented with 100 µm iron chelator Dip and separated into membrane and supernatant subcellular fractions. The subcellular distribution of RhuH was evaluated by immunoblotting using polyclonal anti‐RhuH antibodies. RhuA is a control for a membrane protein. M: Molecular weight; Lane 1: rRhuH; Lane 2: cell lysate of RA CH‐1; Lane 3: cell lysate of *ΔrhuH*; Lane 4: cell lysate of *ΔrhuH* ^C^; Lane 5: extracted secretion protein from RA CH‐1; Lane 6: extracted secretion protein from *ΔrhuH*; Lane 7: extracted secretion protein from *ΔrhuH^C^*. b) TEM images of RA CH‐1 in the stationary phase. The arrow indicates OMVs secreted from RA CH‐1. Scale bars = 200 nm. c) TEM images of OMVs. The arrows show five OMVs of RA CH‐1. Scale bars = 100 nm. d) The distribution of the proteins in OMVs and OMVs‐Dip was evaluated by immunoblotting using the corresponding polyclonal antibodies. M: Molecular weight. Lane 1: rRhuH. Lane 2: rRhuA. Lane 3: rRecA. Lane 4: OMVs of RA CH‐1. Lane 5: OMVs‐Dip of RA CH‐1.

To understand the role of RhuH in haem utilization, we sought to determine the subcellular localization of RhuH. Sequence analysis showed that RhuH contains an N‐terminal signal peptide (1–19 amino acids) and the absence of a membrane‐anchoring signal, indicated that RhuH could be surface‐exposed or secreted. We did not detect RhuH in the whole‐cell extracts of RA CH‐1, even when cultured in an iron‐limited medium. Therefore, we hypothesized that RhuH was secreted into the medium. To test this, *R. anatipestifer* CH‐1 was grown in GCB and GCB with iron limitation, and secreted proteins were assessed. When analyzed by western blot, RhuH was found to be mainly secreted into extracellular media in iron‐limited conditions (**Figure** **2**a). As a control, a western blot with antibodies against the outer membrane–exposed protein RhuA was used to ensure the correct fractionation of *R. anatipestifer* cultures. This showed that RhuA is mainly distributed in the membrane fraction and is secreted in iron‐limited conditions, but is only detected in the membrane fraction in iron‐rich conditions (Figure 2a). Overall, these results demonstrated that RhuH was secreted into the extracellular environment.

Gram‐negative bacteria produce OMVs during their growth process, and one of the functions of OMVs is nutrient acquisition.^[^ ^23^ ^]^ In Gram‐positive bacterium *Dietzia* sp. DQ12‐45‐1b, *Dt*HtaA was found to be distributed in membrane vesicles (MVs) and capable of binding both free haem and haem from hemoglobin.^[^ ^24^ ^]^ Therefore, we identified whether *R. anatipestifer* produces OMVs and whether RhuH was secreted within these. Transmission electron microscopy (TEM) observations revealed the presence of budding vesicles on the surface of *R. anatipestifer* CH‐1 cells (Figure 2b). Subsequently, we used ultracentrifugation to separate and extract OMVs and observed five OMVs through negative staining and TEM (Figure 2c). This result indicates that *R. anatipestifer* can secrete OMVs during its growth process.

We used western blotting to detect the presence of RhuH in OMVs. Since RhuH was detected in the secretions of RA CH‐1 cultured under iron‐limited conditions, we extracted OMVs from RA CH‐1 cultured under the GCB condition, as well as from GCB containing 100 µm 2,2′‐dipyridyl (Dip) condition (OMVs‐Dip). OMVs and OMVs‐Dip were separated via SDS‐PAGE and subsequently transferred to nitrocellulose membranes for detection using specific antibodies. RhuH was not detected in OMVs, whereas this was detectable in OMVs‐Dip (Figure 2d). Furthermore, RhuA was also detected in OMVs‐Dip. These results suggest that under iron‐restricted conditions, both RhuH and RhuA are secreted into the extracellular environment in OMVs.

### 2.3 RhuH is a Haem Binding Protein

**Figure 3:**
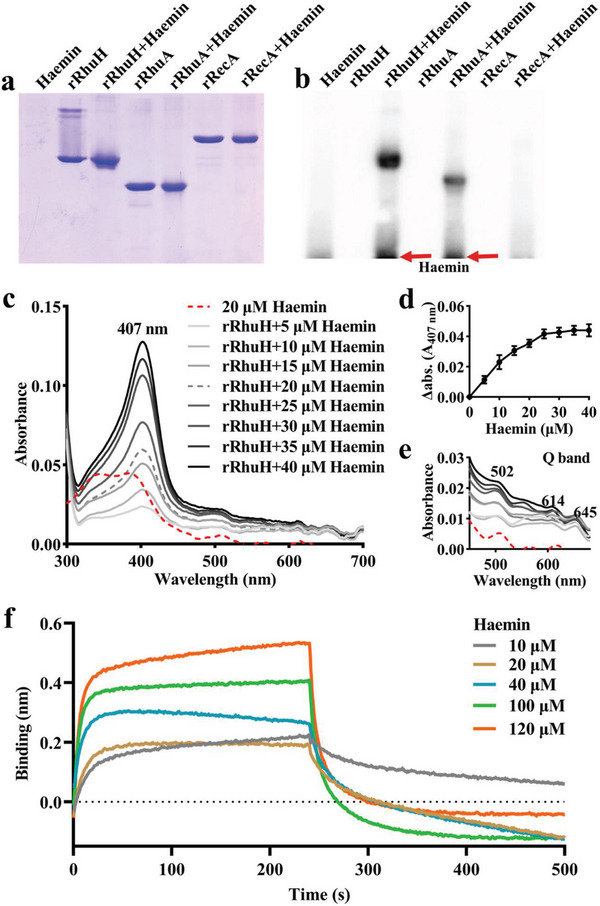
Recombinant RhuH binds haem. a,b) Coomassie blue staining (a) and ECL detaction (b) of haemin binding by rRhuH. Red arrows display free haemin. c) Absorption spectra of haemin binding to apo‐rRhuH. Titration of haemin (0 to 40 µm) to apo‐rRhuH (20 µm). The spectrum of 20 µm haemin alone (red dashed line) in 340 to 385 nm range, whereas the spectral lines ranging from light gray to black correspond to rRhuH incubated with haemin, demonstrating an increase in peak height as haemin concentrations are elevated. d) The change in absorbance (Δ abs.) at 407 nm for apo‐rRhuH bound to haemin at increasing concentrations. e) Changes in the Q bands. Experiments were performed in triplicate, and the results of a single representative experiment are presented. f) Biolayer interferometry (BLI) determination of the binding kinetics between the rRhuH and haemin. BLI response profile of haemin at different concentrations (10–120 µm) with the sensor‐immobilized rRhuH, resulting in a dissociation constant (*K*_d_) of 3.44 × 10^−11^ m.

RhuH without the predicted signal peptide was expressed recombinantly with a His‐tag. We purified the recombinant mature RhuH protein (lacking the signal peptide) from *Escherichia coli*. Based on its sequence has a molecular weight of 35 kDa, whereas eluate at 44.5–76 kDa range from the size exclusion chromatography corresponding to a dimer, and the signal at 410 nm proves that the purified protein is an apo protein (Figure S1a,b, Supporting Information). To confirm that RhuH binds haem, we performed two haem binding assays to determine its haem binding capabilities. The recombinant RhuH protein (rRhuH), recombinant RhuA protein (rRhuA) (positive control),^[^ ^20a^ ^]^ recombinant RecA protein (rRecA) (negative control),^[^ ^20a^ ^]^ and the complexes of recombinant protein incubated with haemin were evaluated via SDS‐PAGE followed by Coomassie blue staining (**Figure** **3**a) or western blotting detected by the enhanced chemiluminescence (ECL) method (Figure 3b).^[^ ^13a^ ^]^ The haemin signal was detected in the haem binding protein rRhuA (positive control) incubated with haemin and in rRhuH incubated with haemin, although the rRecA (negative control) incubated with haemin was not detectable (Figure 3b).

The haem‐binding capacity of rRhuH vas determined via absorption spectroscopy measurement where free haemin has two peak in the 340–385 nm range. Recombinant RhuH (20 µm) was incubated with haemin (1–40 µm), producing a Soret peak at 407 nm (Figure 3c), suggesting that a RhuH–haemin complex was formed.^[^ ^25^ ^]^ The titration of haemin to rRhuH via differential absorption spectroscopy at 407 nm produced a stoichiometry of 1:1 of haemin to rRhuH (Figure 3d). Soret peaks of Q band appear at 502, 614, and 645 nm (Figure 3e). Biolayer interferometry (BLI) assays were performed to evaluate the affinity of RhuH to haemin, and the results showed that RhuH bound to haemin with a dissociation constant (*K*_d_) of 3.44 × 10^−11^ m (Figure 3f). These results indicate that RhuH can bind haem with high affinity.

### 2.4 Recombinant RhuH Could Extract Haem from Duck Hemoglobin

**Figure 4:**
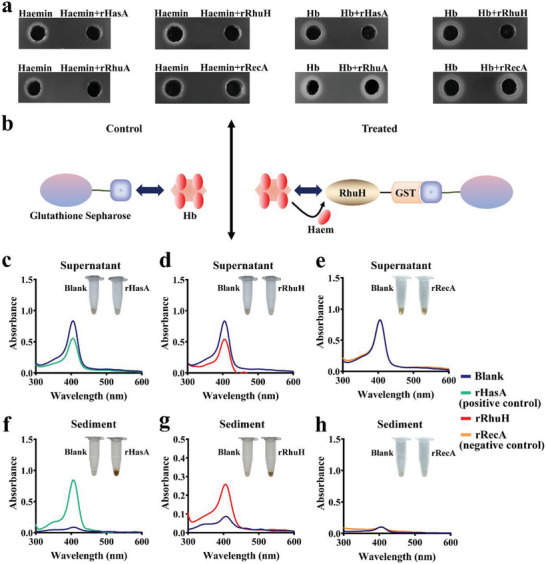
rRhuH could capture haem from duck hemoglobin. a) *E. coli* C600*ΔhemA* pAM238*::hemR* was mixed with 4 mL of soft LB agar and poured onto LB plates. Wells were cut into LB plates, and 20 µm haemin, a mixture of 20 µm haemin with 20 µm recombinant protein, and 5 µm duck Hb, and a mixture of 5 µm duck Hb with 20 µm recombinant protein were added. rHasA, recombinant HasA of *S. marcescens*; rRhuH, recombinant RhuH of *R. anatipestifer* CH‐1. rRhuA, recombinant RhuA of *R. anatipestifer* CH‐1; and rRecA, recombinant RecA of *R. anatipestifer* CH‐1. Plates were incubated at 37 °C overnight. The experiment was repeated in triplicate, and a representative result is presented. The lucid zone represents the zone of C600 *ΔhemA* pAM238*::hemR* growth. b) Diagram of haem transfer assay. c–h) Glutathione‐sepharose loaded with 20 µm GST‐rRhuH, GST‐rHasA (positive control), GST‐rRecA (negative control), or PBS (blank) was treated with 5 µm duck hemoglobin for 30 min. Samples were centrifuged and separated into supernatant (c–e) and sediment (f–h), followed by absorption spectroscopy measurement. Insets display the haem pigment change with GST‐rRhuH, GST‐rHasA, GST‐rRecA, or GST (blank) treated.

Since most of haem in vivo is present in forms of haemoproteins, expescially host hemoglobin, led us to investigate if rRhuH scavenges haem from duck hemoglobin. Consequently, we used the haem utilization model *E. coli* strain C600*ΔhemA* pAM238*::hemR*, which is effective in testing haem binding and extraction.^[^ ^20a^ ^]^ C600*ΔhemA* pAM238*::hemR* could only grow on the Luria‐Bertani (LB) plate supplemented with haemin or hemoglobin, since HemR can transport haem into the cell.^[^ ^26^ ^]^ If the extracellular haem is bound by other haem binding proteins, this will prevent haem transport by HemR and inhibit the growth of C600*ΔhemA* pAM238*::hemR*.^[^ ^20a^ ^]^ Similar to rHasA, which can bind and extract haem from duck hemoglobin (Hb),^[^ ^20a^ ^]^ rRhuH prevented HemR from utilizing haemin and duck hemoglobin (**Figure** **4**a). As a control, the rRhuA only prevented HemR from using haemin but not duck hemoglobin. The rRecA did not affect the haemin and hemoglobin use of C600*ΔhemA* pAM238*::hemR*. Therefore, we predict that rRhuH could extract haem from duck hemoglobin.

Next, we used a protocol described by Maresso^[^ ^27^ ^]^ with a minor modification to examine the haem extracted from duck hemoglobin. Glutathione‐sepharose resin loaded with GST‐rRhuH, GST‐rHasA or GST‐rRecA (20 µm) was incubated with 5 µm duck hemoglobin (Hb) (Figure 4b). The resin was centrifuged, washed, and the GST‐rRhuH or GST‐rHasA eluted; the supernatant containing duck hemoglobin was then separated (Figure 4b). As a control, 5 µm duck hemoglobin was incubated with glutathione‐sepharose and PBS complexes and compared with GST‐rRhuH–, GST‐rHasA– or GST‐rRecA–treated samples (Figure 4b). After incubation with GST‐rHasA (positive control), the haem‐specific absorbance of duck hemoglobin at 413 nm was decreased, similarly, the absorbance of duck hemoglobin incubated with GST‐rRhuH at 413 nm was also decreased, indicating that rRhuH had removed haem from duck hemoglobin (Figure 4c,d). After incubation with GST‐rRecA (negative control), the haem‐specific absorbance of duck hemoglobin at 413 nm remained unchanged (Figure 4e). Removal of haem can also be observed by the color change of duck hemoglobin. The red‐brown color of duck hemoglobin is faded by GST‐rHasA or GST‐rRhuH treatment, but not by the untreated sample or GST‐rRecA treatment (inset, Figure 4c–e). Then, the eluted protein was analyzed by spectrophotometry. Both GST‐rHasA and GST‐rRhuH displayed an increase in absorbance at 407 nm after incubation with duck hemoglobin (Figure 4f,g), while GST‐rRecA showed no significant absorption peak (Figure 4h). The glutathione‐sepharose sediment displayed red‐brown pigmented GST‐rHasA and GST‐rRhuH, while untreated sample or GST‐rRecA treatment remained clear (inset, Figure 4f–h). Taken together, all these results indicated that recombinant RhuH was able to extract haem from duck hemoglobin.

### 2.5 Crystal Structure of apo‐RhuH

**Figure 5:**
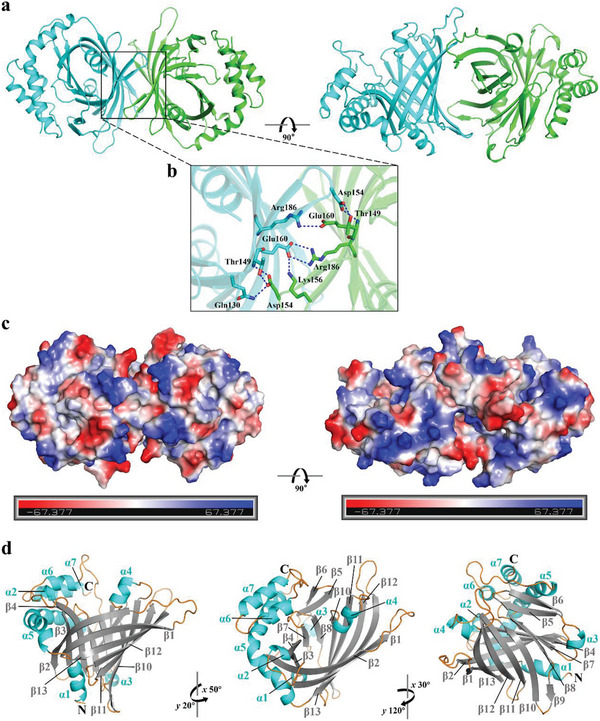
3D structure of RhuH. a and b) The dimeric structure of RhuH from different perspectives, with an expanded view of interactions at the dimer interface between the two RhuH monomers. The interaction residues (shown as sticks in blue and green by elements, respectively) interact via hydrogen bonds (indicated by blue dashed lines) (b). c) Electrostatic surface potential of RhuH protein. Positively charged regions are denoted in blue while negatively charged areas are represented in red. d) Monomeric structure of RhuH from three different orientations, comprising seven α‐helices (shown in blue), a β‐hairpin formed by β5 and β6 (shown in gray), and a β‐barrel‐like structure formed by β1–β4 and β7–β13 (shown in gray); loops are shown in orange.

To gain insight into the role of RhuH, we solved the X‐ray crystal structure of it at 2.85 Å resolution (PDB ID: 8XJC). Details on crystallographic data collection and structural refinement statistics are provided in Table S3 (Supporting Information). The crystal structure of RhuH reveals a homodimer (**Figure** **5**a), each subunit containing an N‐terminal uncompact barrel‐like domain (residues 37–226), which is partially surrounded by a more flexible C‐terminal domain (residues 227–298) consisting of loops and several α‐helices situated on one side, resulting in an asymmetric structure. β8 to β10 are major regions of interaction in the homodimerization of RhuH by forming several intersubunit hydrogen bonds, including Gln130, Thr149, Asp154, Arg186, and Glu160 from one monomer, and Asp154, Thr149, Arg186, Glu160, and Lys156 from the other monomer (Figure 5b). The electrostatic surface potential of the dimer reveals the charge distribution of the protein structure (Figure 5c). As shown in Figure 5d, the monomeric structure of RhuH comprises seven α‐helices while a β‐hairpin is formed by β5 and β6 strands and features an 11‐stranded β‐barrel‐like structure, which comprises eleven antiparallel β‐strands (β1‐β4, β7‐β13) with noncontinuous residues, two short loops and four long extensions on the same side (Figure 5d). The protein structure of RhuH distinctively differs from the determined structures of other haemophores (Figure S2a–f, Supporting Information), including HasA (PDB:1DKH) in *S. marcescens*,^[^ ^28^ ^]^ HmuY (PDB:6EWM)^[^ ^18^ ^]^ and HusA (PDB:6BQS)^[^ ^16^ ^]^ in *P. gingivalis*, HxuA (PDB: 4RM6) in *H. influenzae*,^[^ ^9^ ^]^ and HphA (PDB:7REA) in *A. baumannii*.^[^ ^11^ ^]^

### 2.6 Key Amino Acid Residues for Dimer Formation and Haem Extraction

In the amino acid residues formed by the dimerization of RhuH, the Asp154 (154D) or Glu160 (160E) residue of one monomer formed hydrogen bonds with two residues of another monomer (Figure 5b), suggesting that these two residues may play a role in the formation and stability of the dimer. Therefore, these two sites were individually and collectively mutated to alanine (ALA) to assess the impact on dimer formation. The recombinant proteins were separated by SDS‐PAGE and stained with Coomassie brilliant blue (Figure S3a, Supporting Information). Recombinant proteins rRhuH and rRhuH^154D‐A^ exhibited double bands, whereas the rRhuH^160E‐A^ and rRhuH^154D‐A‐160E‐A^ exhibited only a single band, indicating that Glu160 is a key amino acid for dimer formation. To identify whether the mutation of this critical dimer site affects haem utilization, we evaluated the growth curve of the WT and mutants in GCB medium using duck hemoglobin or haemin as the sole iron source. The growth of the RA CH‐1 *rhuH^160E‐A^* (*rhuH^160E‐A^*) strain was the same as the WT strain, whereas that of the *ΔrhuH* strain was significantly delayed (Figure S3b,c, Supporting Information). Absorption spectroscopy measurement showed that the binding levels of rRhuH and rRhuH^160E‐A^ to haemin did not significantly differ (Figure S3d, Supporting Information). These results indicate that the mutation of the critical dimer site results in a monomeric form of RhuH while its ability to utilize haem remains unaffected.

**Figure 6:**
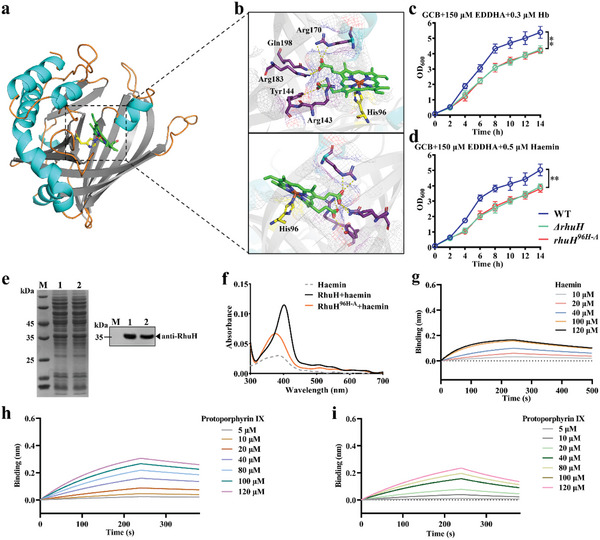
Haem iron coordinated His96 is a critical haem binding site. a) The structure of the RhuH–haem complex (holo‐RhuH) predicted by Alphafold3 is shown as a cartoon representation, revealing that the haem binding pocket resides in the β‐barrel‐like region. Haem is shown as sticks in green by element. b) An expanded view of the RhuH binding to haem. His96 coordinates with the haem iron and is shown as stick in yellow by element. Arg170, Arg183, Arg143, Tyr144, and Gln198 are linked to haem propionate groups by hydrogen bonds and are shown as sticks in purple by element. Hydrogen bonds are represented by yellow dashed lines. The predicted template modeling (pTM) score by Alphafold3 is 0.93, and the interface‐predicted template modeling (ipTM) score is 0.86, with pLDDT >90. c and d) Growth curves of WT, *ΔrhuH*, and the RA CH‐1 *rhuH^96H‐A^* (*rhuH^96H‐A^*) mutant strains under iron‐limited GCB supplemented with 0.3 µm duck Hb (c) or 0.5 µm haemin (d). Data represents mean plus standard deviations from three independent experiments each in biological triplicate. Statistical analyses were conducted using unpaired *t*‐test. ^**^, *p *< 0.01. e) Western blotting was employed to assess the expression of RhuH in the secreted proteins of RA CH‐1 and point mutation strains. M: molecular weight; Lane 1: extracted secretion protein from RA CH‐1; Lane 2: extracted secretion protein from RA CH‐1 *rhuH^96H‐A^*. f) Absorption spectra of 20 µm RhuH or RhuH^96H‐A^ binding to 20 µm haemin. g) Biolayer interferometry (BLI) determination of binding kinetics between rRhuH^96H‐A^ and haemin. BLI response profile of haemin at different concentrations (10–120 µm) with the sensor‐immobilized rRhuH^96H‐A^, producing a dissociation constant (*K*_d_) of 2.022 × 10^−5^ m. h–i) BLI determination of the binding kinetics between rRhuH or rRhuH^96H‐A^ and protoporphyrin IX. BLI response profile of protoporphyrin IX at different concentrations (5–120 µm) with the sensor‐immobilized rRhuH (h) or rRhuH^96H‐A^ (i). The resulting dissociation constants (*K*_d_) are 2.714 × 10^−5^ m for rRhuH (h) and 4.031 × 10^−5^ m for rRhuH^96H‐A^ (i), respectively.

Next, we identified the key amino acid residues for haem extraction. We attempted to elucidate the complex structure of RhuH and haem using X‐ray crystallography. Unfortunately, we could not obtain a crystal structure of the complex after many attempts. Therefore, we used the AlphaFold3 online tool to predict the complex structure.^[^ ^29^ ^]^ First, we compared the RhuH protein structure predicted by Alphafold3 with its crystal structure. The overall structures were similar, with only slight differences in some of the more flexible loops, indicating that the Alphafold3 prediction of the RhuH structure is highly accurate (Figure S4, Supporting Information). Subsequently, the RhuH–haem complex structure, namely holo‐RhuH, was predicted using Alphafold3. As shown in **Figure** **6**a,b, RhuH coordinates the haem iron by His96 on the proximal side, while Arg170, Arg183 and Arg143, tyrosine Tyr144, and Gln198 form hydrogen bonds with haem propionate groups. To confirm the role of His96 in haem binding, the corresponding site in the genome of RA CH‐1 was mutated to alanine (A), resulting in the RA CH‐1 *rhuH^96H‐A^* point‐mutant strain. Subsequently, we compared the ability of the WT and this mutant strain to utilize duck hemoglobin or haemin in iron‐limited conditions. When 0.3 µm duck hemoglobin or 0.5 µm haemin was the sole iron source, growth was significantly delayed in the *ΔrhuH* and RA CH‐1 *rhuH^96H‐A^* (*rhuH^96H‐A^*) strains compared with that in the WT strain (Figure 6c,d). To validate whether site‐directed mutations affect the normal expression of RhuH in the RA CH‐1 strain, we performed western blot analysis using antibodies specific to RhuH. The RhuH protein was detectable in secretions from the *rhuH^96H‐A^* strain (Figure 6e), indicating that site mutations did not affect protein expression. This indicates that His96 is involved in the use of haem.

We then used absorption spectroscopy to determine the haem binding capacity of the rRhuH^96H‐A^ mutant. Compared with rRhuH, rRhuH^96H‐A^ displayed a significantly reduced ability to bind haemin (Figure 6f). BLI assays showed that RhuH^96H‐A^ bound to haemin with a dissociation constant (*K*_d_) of 2.022 × 10^−5^ m (Figure 6g). Compared to the *K*_d_ value of RhuH binding to haemin (3.44 × 10^−11^ m), the binding affinity had significantly decreased by approximately six orders of magnitude. Since the haem propionate groups interacted with intramolecular residues via hydrogen bonds, we aimed to determine whether the binding of RhuH to haem is primarily mediated by haem iron coordination or hydrogen bonding. Consequently, we employed a BLI assay to measure the affinity of the protein for protoporphyrin IX and demonstrated that RhuH and RhuH^96H‐A^ exhibited comparable binding affinities of *K*_d_ of 2.714 × 10^−5^  and 4.031 × 10^−5^ m when bound to protoporphyrin IX, respectively (Figure 6h,i). Moreover, the affinity of RhuH for protoporphyrin IX was approximately six orders of magnitude lower than that for haem. These results collectively suggest that the binding of RhuH to haem predominantly relies on haem iron coordination and that the His96 residue in RhuH is essential for haem binding.

### 2.7 Inactivation of *rhuH* does not Affect Pathogenicity of *R. anatipestifer* CH‐1

To assess the relevance of *rhuH* to bacterial pathogenicity in vivo, duckling infection models were performed. Lethal doses of *R. anatipestifer* strains CH‐1, *ΔrhuH* and *rhuH^96H‐A^* were prepared to infect three‐day‐old ducklings, as described in detail in the supplementary materials and methods. As shown in Figure S5a (Supporting Information), the survival rate of ducklings infected with the *ΔrhuH* strain or the *rhuH^96H‐A^* mutant strain was similar to those infected with the WT strain. Subsequently, the colonization level of the WT, *ΔrhuH* and *rhuH^96H‐A^* was determined at 24 h postinfection. The result showed that the bacterial load of the *ΔrhuH* and *rhuH^96H‐A^* in blood, spleen, liver, and brain was not significantly different from that of the WT strain (Figure S5b, Supporting Information). These results suggest that the *rhuH* deletion or mutation does not affect the pathogenicity of *R. anatipestifer* CH‐1.

### 2.8 Distribution of RhuH Homologues

**Figure 7:**
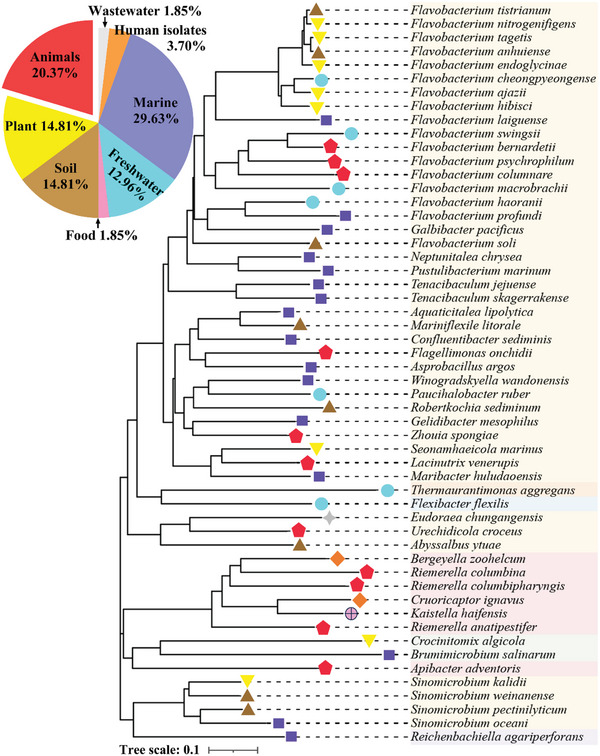
Phylogenetic tree of RhuH and homologous proteins. The tree distance is the calculated distance from the multiple sequence alignment in the MEGA7 program, using the neighbor joining method. The scale bar indicates the percentage of divergence (distance). Species were isolated from soil (14.81%, brown triangle), animal (20.37%, red pentagon), marine (29.63%, blue square), freshwater (12.96%, cyan circle), humans (3.70%, orange rhombus), food (1.85%, pink circle), and wastewater (1.85%, grey star).

Sequence comparison analysis showed that RhuH had low homology to the characterized haemophore HasA of *S. marcescens* (8.05% identity), HusA (10.7% identity) and HmuY (7.69% identity) of *P. gingivalis*, HxuA of *H. influenzae* (4.75% identity), and HphA of *A. baumannii* (9.4% identity). A BLASTP search showed that RhuH homologues are present in all complete *R. anatipestifer* genomes, with 97.9%–99.6% sequence identity. The orthologs of RhuH are also present in the *Flavobacteriales* order, including *Weeksellaceae* and *Flavobacteriaceae*, with sequence similarity >50% and coverage > 90%, including *Bergeyella zoohelcum*, *Riemerella columbina*, and *Flavobacterium profundi*, which are mainly distributed in animals (20.37%), marine (29.09%), soil (14.55%), freshwater (12.73%), and plant (14.55%) environments (**Figure** **7**). The RhuH phylogenetic tree was constructed using the neighbor joining algorithm by MEGA 7 program, providing insight into the evolutionary conservation of this novel haemophore.

### 2.9 The Homologues of RhuH have Conserved Function in Other Members of the *Flavobacteriales*

**Figure 8:**
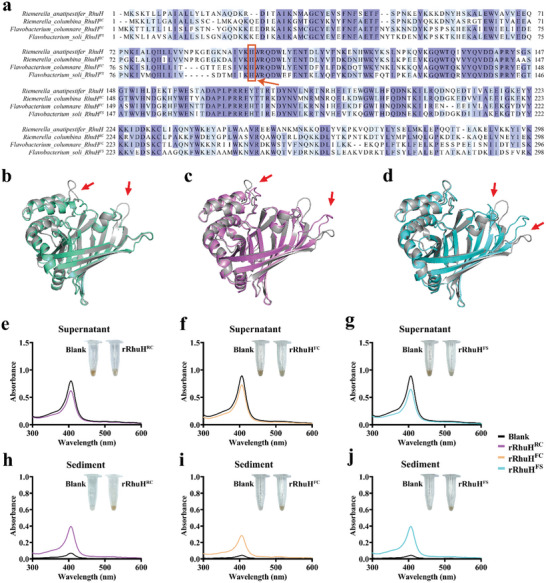
The homologous protein RhuH^RC^, RhuH^FC^, and RhuH^FS^ show high similarity to RhuH in structure and function. a) Multiple sequence alignment of RhuH and its homologous proteins. Amino acid sequences of RhuH from *R. anatipestifer*, RhuH^RC^ from *R. columbina*, RhuH^FC^ from *F. columnare*, and RhuH^FS^ from *F. soli* were aligned using the ClustalW method. Conserved regions were highlighted based on sequence conservation, with an overall sequence identity of 66.61%. The haem‐binding site His96 in RhuH is conserved in RhuH^RC^, RhuH^FC,^ and RhuH^FS^, corresponding to His96, His97, and His95, respectively. These residues are marked with red boxes and indicated by arrows. b–d) The structures of RhuH^RC^ (a, green), RhuH^FC^ (b, purple), and RhuH^FS^ (c, cyan) were constructed using AlphaFold3. These structures were compared with RhuH (PDB ID: 8XJC), shown in gray. The overall structures of RhuH^RC^, RhuH^FC^, and RhuH^FS^ are largely similar to RhuH, with minor differences in some loops, as indicated by the arrows. e–j) Glutathione‐sepharose loaded with 20 µm GST‐rRhuH^RC^, GST‐rRhuH^FC^, GST‐rRhuH^FS^, or PBS (blank) was treated with 5 µm bovine hemoglobin for 30 min. Samples were centrifuged and separated into supernatant (e–g) and sediment (h–j), followed by absorption spectroscopy measurement. Insets display the haem pigment change with GST‐rRhuH^RC^, GST‐rRhuH^FC^, GST‐rRhuH^FS^, or GST (blank) treated. In this experiment, e and h were performed first, with its own blank group, while groups f and g, i, and j were conducted together, sharing the same blank group.

To further assess the biological function of RhuH homologues in other members of the *Flavobacteriales*, the structure and function of RhuH homologs in *Riemerella columbina* (*R. columbina*), *Flavobacterium columnare* (*F. columnare*), and *Flavobacterium soli* (*F. soli*) which share 64.21%, 50.5% and 50.83% amino acid sequence identity with RhuH, respectively, were identified. Here, the RhuH homologs in *R. columbina*, *F. columnare* and *F. soli* were designated as RhuH^RC^, RhuH^FC^, and RhuH^FS^, respectively. Sequence alignment confirmed that the critical haem‐binding residue His96 in RhuH is highly conserved in RhuH^RC^, RhuH^FC,^ and RhuH^FS^, corresponding to His96, His97, and His95, respectively (**Figure** **8**a). Structural comparisons were performed using protein models of RhuH^RC^, RhuH^FC^, and RhuH^FS^ predicted by the Alphafold3 online tool. As shown in Figure 8b–d, the structures of RhuH^RC^, RhuH^FC^, and RhuH^FS^ are highly similar to RhuH, with differences observed primarily in flexible loop regions.

Subsequently, we assessed the ability of recombinant RhuH^RC^, recombinant RhuH^FC,^ and recombinant RhuH^FS^ to capture haem from bovine hemoglobin. As shown in Figure 8e–g, incubation with GST‐rRhuH^RC^, GST‐rRhuH^FC^ or GST‐rRhuH^FS^ resulted in a decrease in the haem‐specific absorbance of bovine hemoglobin at 406 nm, accompanied by a fading of its red–brown color, which remained unchanged in untreated controls (insets, Figure 8e–g). Further spectrophotometric analysis revealed increased absorbance at 407 nm for GST‐rRhuH^RC^, GST‐rRhuH^FC^, and GST‐rRhuH^FS^ after incubation with bovine hemoglobin (Figure 8h–j). Glutathione‐Sepharose sediment showed red–brown pigmented GST‐rRhuH^RC^, GST‐rRhuH^FC^ or GST‐rRhuH^FS^, while untreated samples remained clear (insets, Figure 8h–j). These results demonstrated that recombinant RhuH^RC^, RhuH^FC^, and RhuH^FS^ can extract haem from bovine hemoglobin. In summary, these results indicated that the homologues of RhuH in other bacterial species are highly conserved in both structure and function, and may play an important biological role in haem acquisition by bacteria within the order *Flavobacteriales*.

## 3 Discussion

To establish an infection in the vertebrate host, bacteria must acquire iron from the host. However, large amounts of iron are present as haem, which is primarily bound to hemoglobin in red blood cells.^[^ ^30^ ^]^ Many bacterial pathogens have evolved mechanisms to obtain iron through the transport of haem from hemoglobin. The haem transport system in Gram‐negative bacteria mainly comprises haemophores, TonB‐dependent receptors, and the TonB‐ExbB‐ExbD complex. and in some cases, extracellular haem‐binding protein, like haemophores, are needed to more efficiently obtain haem from haemoproteins. Haemophores are types of proteins that are secreted into the extracellular environment and can acquire haem from haemoproteins, like hemoglobin.^[^ ^31^ ^]^ Haem is then passed to the TonB‐dependent haem receptor and transferred into the periplasm via the proton motive force provided by the TonB‐ExbB‐ExbD complex, and finally to the cytoplasm by an ABC transporter.^[^ ^3b^ ^]^ Currently, five classical haemophores have been characterized in Gram‐negative bacteria. The HasA monomeric structure of *S. marcescens* comprises an extended antiparallel β‐sheet and a four α‐helix wall, from which two loops extrude to sandwich the haem molecule (Figure S2b, Supporting Information).^[^ ^12b^ ^]^ The haem iron is axially coordinated by His32 and Tyr75, which also engages in a hydrogen bond interaction with His83.^[^ ^12b^ ^]^ HxuA of *H. influenzae* is an elongated molecule that adopts a right‐handed β‐helix conformation with three parallel β‐sheets (Figure S2e, Supporting Information). HxuA is inserted into the middle of the two subunits of haemopexin, redirecting key residues in the haem‐binding pocket of haemopexin, causing haem ejection.^[^ ^9^ ^]^ HusA of *P. gingivalis* comprises nine α‐helices, organized into four helix‐turn‐helix motifs, with an additional C‐terminal helix. Together, these form a right‐handed superhelical arrangement characterized by a concave and a convex surface (Figure S2d, Supporting Information).^[^ ^16^ ^]^ Haem is bound within a hydrophobic groove on the α‐helical structure of HusA, with a high affinity *K*_d_ of 7.0 ± 2.5 × 10^−10^ m. This binding configuration is distinct from the common iron coordination found in other haemophores.^[^ ^16^ ^]^ HmuY of *P. gingivalis* mainly comprises β‐structures (Figure S2c, Supporting Information) and can bind haem by utilizing the histidine residues His134 and His166 to coordinate with haem iron.^[^ ^10a^ ^]^ HmuY family proteins sometimes also coordinate the haem iron by two methionine residues, such as Met162 and Met191 in *Prevotella intermedia* PinA,^[^ ^32^ ^]^ Met143 and Met169 in *Tannerella forsythia* Tfo^[^ ^18^ ^]^ and Met145 and Met172 in *Bacteroides vulgatus* Bvu.^[^ ^33^ ^]^ HphA of *A. baumannii* comprises a C‐terminal eight‐stranded β‐barrel, six intermediate β‐strands, and an N‐terminal clamp‐like structure (Figure S2f, Supporting Information).^[^ ^11^ ^]^ Two histidine residues within HphA, His43 and His106, coordinate with the haem iron to enable the extraction of haem from both hemoglobin and serum albumin.^[^ ^11^ ^]^

Herein, we discovered that the structure of the haemophore RhuH is distinct from the structures of the aforementioned haemophores. Most α‐helices are located on one side of the molecule while the β‐hairpin and a β‐barrel‐like structure are positioned on the other side. The β‐barrel structure of RhuH is similar to the eight‐stranded β‐barrel structure in HphA of *A. baumannii*, which contains contiguous amino acids.^[^ ^11^ ^]^ However, in RhuH, the 11‐stranded β‐barrel‐like structure contains discontinuous amino acids. The haem binding pocket of holo‐RhuH is in the β‐barrel‐like structure region, with the haem molecule parallel to the β4 strand and α3 helix. The haem iron is coordinated by His96, and the haem propionate groups are oriented toward the β‐barrel‐like structure, buried within the molecule to form hydrogen bonds with internal amino acid residues, thus stabilizing the haem. This orientation of the haem is similar to that observed in the HmuY‐haem complex, where the haem propionate groups are buried within the molecule,^[^ ^10a^ ^]^ and contrasts with that in the holo‐HasA and holo‐HphA, where the haem propionate groups are exposed to the solvent.^[^ ^11^, ^34^ ^]^ In other haemophores, one known case also has only one residue that coordinates with the haem iron in holo‐HasA of *Yersinia pestis*, which coordinates with the haem iron through a conserved tyrosine residue.^[^ ^35^ ^]^ We speculate that in the holo‐RhuH, the metal coordination bond and hydrogen bonds may synergistically act to contribute to the stability of the haem within the protein molecule.

In this study, RhuH was crystallized as a dimer. Other haemophores also exist in dimeric or even multimeric forms. For example, HasA from *S. marcescens* can form a dimeric structure (DHasA) through domain swapping, a structural mechanism in which two or more identical protein molecules exchange parts of their structure, forming a stable dimer or oligomer.^[^ ^28^ ^]^ In DHasA, the domain‐swapping interaction involves the exchange of a domain containing one of the haem ligands, His32, between two polypeptide chains.^[^ ^28^ ^]^ This structural arrangement allows DHasA to bind two haem. However, DHasA cannot directly transfer haem to the HasR, instead it efficiently transfers haem to the monomeric form of HasA, which then transfers this to HasR.^[^ ^28^ ^]^ Thus, DHasA could act as a haem reservoir in the haemophore system. HmuY of *P. gingivalis* exists as a cross‐like tetrameric complex, and when haem binds to HmuY, this transitions from a dimer to a tetramer, which helps protect haem from host scavengers and shepherds this toward HmuR.^[^ ^10a^ ^]^ Therefore, we speculate that the dimeric structure of RhuH may be more advantageous in binding and storing haem.

HasA can bind haem with a high affinity (*K*_d_ = 1.8 × 10⁻¹¹ m), and the process of acquiring haem from hemoglobin is therefore considered as passive rather than direct extraction.^[^ ^36^ ^]^ HphA also obtained haem through a passive process, possibly because of its high affinity for haem.^[^ ^11^ ^]^ Conversely, HmuY has a lower affinity for haem (*K*_d_ <10⁻⁹ m), ensuring an efficient haem supply through its correlation with gingipains Kgp/RgpA, where Kgp digests haemoproteins to release haem while RgpA participates in the oxidation of oxyhemoglobin to methemoglobin, facilitating haem release.^[^ ^37^ ^]^ Herein, RhuH could acquire haem from host hemoglobin, and bind haem with high affinity (*K*_d_ = 3.44 × 10⁻¹¹ m). Therefore, we speculate that RhuH acquires haem through a high affinity passive process.

In this study, RhuH is secreted as a protein in OMVs. Based on previous research,^[^ ^24^ ^]^ we proposed two possible mechanisms for the function of RhuH secreted by OMVs. The first possibility is that OMVs undergo lysis in the environment, releasing RhuH, which then captures haem from host hemoglobin. The haem is subsequently transferred to the appropriate outer membrane receptor and transported into the cell for bacterial utilization. The second possibility is that RhuH is located on the surface of OMVs, where it acquires haem directly from host hemoglobin in the environment. In this case, OMVs then transport the haem into the cell through membrane fusion.

Previously we showed that the outer membrane protein RhuA and the TonB2‐dependent haem receptor RhuR allow haem entry into the bacterial cell, but RhuA could not acquire haem from host hemoglobin.^[^ ^20a^ ^]^ Herein, the identification of the haemophore RhuH suggests that haem acquired by RhuH is transferred to RhuA or RhuR, thereby facilitating haem transfer into the cell. Therefore, our subsequent research will focus on the relationship between RhuA, RhuR, and RhuH to gain a deeper understanding of the haem uptake system in *R. anatipestifer*. Additionally, the inactivation of *rhuH* does not affect the virulence of *R. anatipestifer* CH‐1, indicating that this bacterium possesses abundant haem utilization systems that remain to be identified. Consistently, the inactivation of *rhuA‐rhuR* or *rhuB*, which encodes a TonB‐dependent haem receptor, did not affect the virulence of *R. anatipestifer* CH‐1.^[^ ^20^, ^38^ ^]^

Overall, RhuH represents a unique haemophore found in the order *Flavobacteriales*. And the functional and structural similarities of the RhuH homologues in *R. columbina*, *F. columnar* *e*, and *F. soli* to RhuH have been demonstrated. The presence of RhuH homologues in bacteria isolated from both mammals and birds, suggests that RhuH homologues may capture haem from different host hemoglobin using a similar strategy. This study is of considerable significance for a deeper comprehension of the haem uptake mechanism in the *Flavobacteriales* bacteria and broadens the understanding of these types of haemophores.

## 4 Experimental Section

### Bacterial Strains, Plasmids, and Primers

The bacterial strains and plasmids used in this study are listed in Table S1 (Supporting Information). All of the primers used in this study are listed in Table S2 (Supporting Information).

### Haemin Binding Assay

Purified recombinant proteins and the complex of proteins and haemin (Sigma, China) were separated via SDS‐PAGE, and then either stained with Coomassie blue or transferred to a nitrocellulose membrane. The membrane was washed with Tris‐buffered saline plus Tween 20 (TBST) (10 mm Tris‐HCl [pH 8.0], 150 mm NaCl, 0.1% Tween 20) for 10 min. Haemin was detected via ECL reagents (Bio‐Rad, USA) in a ChemiDoc MP imaging system (Bio‐Rad, USA).

### BLI‐Binding Kinetics Assay

The binding affinity of RhuH with haem was evaluated using an Octet R2 system (Sartorius) as previously described with minor modifications.^[^ ^39^ ^]^ Briefly, 250 µg mL^−1^ of sample protein was mixed with a specific antibody at a protein‐to‐antibody ratio of 500:1 and incubated overnight at 4 °C. ProA biosensors were hydrated in running buffer (PBS + 0.02% Tween 20) for 10 min, then interacted with the sample protein for 840 s, with a protein immobilization level >4 nm. A series of haemin concentrations (10, 20, 40, 100, and 120 µm) or protoporphyrin IX concentrations (5, 10, 20, 40, 80, 100, and 120 µm) dissolved in running buffer were added to a black polypropylene 96‐well microplate (biosharp, China), with remaining wells filled with PBS. Nonfunctionalized biosensors were used as a control to ensure the absence of nonspecific binding during the assay by measuring all ligand concentrations as well as the running buffer. The assay parameters were a baseline measurement for 60 s, an association phase for 240 s, and a dissociation phase for 300 s. Resulting data were processed using the double reference method in Octet Analysis Studio 13.0, yielding the equilibrium dissociation constant (*K*_d_). Binding curves were exported to GraphPad Prism 9.4 software for curve plotting. The assays were conducted twice for each affinity measurement to ensure reliability.

### rRhuH Extract Haem from Duck Hemoglobin

The haem extraction of RhuH from duck hemoglobin was determined using two methods as previously described.^[^ ^20^, ^27^ ^]^ First, several holes were made in LB plates supplemented with Dip containing *E. coli* C600*ΔhemA* pAM238*::hemR*. Haemin, duck hemoglobin, and the mixture of haemin/duck hemoglobin and recombinant protein were added to the hole, and growth was recorded.

The second method was performed as described by Maresso^[^ ^27^ ^]^ with a minor modification. GST‐rRhuH (20 µm), GST‐rHasA (20 µm, positive control), GST‐rRecA (20 µm, negative control), GST‐rRhuH^RC^ (20 µm), GST‐rRhuH^FC^ (20 µm), GST‐rRhuH^FS^ (20 µm), or PBS (blank) was incubated with 50 µL of glutathione‐sepharose 4B for 90 min at 4 °C, followed by washing three times with 250 µL PBS. Then, 5 µm duck hemoglobin or bovine hemoglobin was added into the samples and incubated for 30 min at room temperature. Samples were centrifuged at 500×*g* for 5 min to separate supernatant (hemoglobin) and sediment (glutathione‐sepharose and GST‐rRhuH, GST‐rHasA, GST‐rRecA, GST‐rRhuH^RC^, GST‐rRhuH^FC^, or GST‐rRhuH^FS^ complexes). The sediment was washed three times with 250 µL PBS, and GST‐rRhuH, GST‐rHasA, GST‐rRecA, GST‐rRhuH^RC^, GST‐rRhuH^FC^, or GST‐rRhuH^FS^ was eluted twice in 25 µL of 600 mm reduced glutathione (pH 8.0). Sediment (GST‐rRhuH, GST‐rHasA, GST‐rRecA, GST‐rRhuH^RC^, GST‐rRhuH^FC^, or GST‐rRhuH^FS^) and supernatant (hemoglobin) were analyzed via spectroscopy using a Nanodrop 2000.

### Crystallization and Structure Determination

Protein samples were crystallized using the sitting drop vapor diffusion method at 16 °C. The crystals appeared in a solution containing 0.2 m Ammonium citrate dibasic and 20% (*w/v*) polyethylene glycol 3350. Harvested crystals were soaked in the reservoir solution supplemented with 25% (*v/v*) glycerol as a cryoprotectant and flash‐frozen in liquid nitrogen. The crystals were collected at the Shanghai Synchrotron Radiation Facility (SSRF), beamline BL‐02U10. Diffraction images were processed with the X‐ray Detector Software (XDS) Program package^[^ ^40^ ^]^ and the structure was determined by the molecular replacement method using the AlphaFold2^[^ ^41^ ^]^‐predicted structure of RhuH as the initial search model. Model building and crystallographic refinement were carried out manually in Coot^[^ ^42^ ^]^ and PHENIX.^[^ ^43^ ^]^ Details of data collection and refinement statistics are provided in, Table S3 (Supporting Information). All figures representing structures were prepared using PyMOL (http://pymol.org).

### Statistical Analysis

Statistical analysis was performed using GraphPad Prism software version 9 (GraphPad Software, CA). Data are presented as mean±standard deviation (SD) from at least three independent experiments (n≥3). An independent Student’ s *t*‐test (two‐tailed) was applied for comparisons between two groups. *P*‐values of less than 0.05 were considered significant.

### Data Availability

The protein sequences of RhuH were deposited in GenBank under accession number WP_014938337.1. Atomic coordinate for the reported crystallographic structure has been deposited at the Protein Data Bank under accession number 8XJC. The GenBank accession numbers of RhuH and its homologues are listed in Supplemental file 1, Table S4. The protein sequences of RhuH^RC^, RhuH^FC^, and RhuH^FS^ was deposited in GenBank under accession number WP_018675666.1, WP_077225713.1, and WP_026706274.1, respectively.

## Conflict of Interest

The authors declare no conflict of interest.

## Author Contributions

M.W., D.Z., and X.T. contributed equally to this work. Author's order was determined by flipping a coin. M.L., A.C., and S.O. conceived and designed the experiments. MY.W, DD.Z., and X.T. performed the experiments. M.L, YM.W., DD.Z., and X.T. wrote the manuscript. J.T., Y.Y, MS.W., DK.Z., and R.J. participated in the experiments. S.C., X.Z., S.Z., J.H., and X.O. analyzed the data. B.T., D.S., Y.H., and Z.W. contributed reagents/materials/analysis tools. All authors have reviewed and approved the manuscript.

## Supporting information



Supporting Information

## Data Availability

The data that support the findings of this study are available in the supplementary material of this article.
