# Supplementary material for: Structural Basis and Mechanism of a Unique Haemophore in the Haem‐Iron Acquisition by Riemerella anatipestifer
Source: Adv Sci (Weinh). 2025 Jan 30;12(13):2412202. doi: 10.1002/advs.202412202 (PMC11967795; doi:10.1002/advs.202412202)
Supplement: Supplementary file 1 — Supporting Information [file ADVS-12-2412202-s001.docx]

Supporting Information

**Structural Basis and Mechanism of a Unique Haemophore in the Haem-Iron Acquisition by** ***Riemerella anatipestifer***

*Mengying Wang^1,2,3,4^**^#^, Dandan* *Zhang^5#^, Xiu* *Tian^1,2,3,4#^, Jiangyang Tong^5^, Yizhou Yao^1,2,3,4^, Mingshu Wang^1,2,3,4^, Dekang* *Zhu^1,2,3,4^, Renyong Jia^1,2,3,4^, Shun Chen^1,2,3,4^, Xinxin Zhao^1,2,3,4^, Shaqiu Zhang^1,2,3,4^, Juan Huang^1,2,3,4^, Xumin Ou^1,2,3,4^, Bin Tian^1,2,3,4^, Di Sun^1,2,3,4^, Yu He^1,2,3,4^, Zhen Wu^1,2,3,4^, Songying Ouyang^5*^, Mafeng Liu^1,2,3,4*^, Anchun* *Cheng^1,2,3,4*^*

1 Engineering Research Center of Southwest Animal Disease Prevention and Control Technology, Ministry of Education of the People's Republic of China, Chengdu 611130, China.

2 Key Laboratory of Animal Disease and Human Health of Sichuan Province, Chengdu 611130, China

3 International Joint Research Center for Animal Disease Prevention and Control of Sichuan Province, Chengdu 611130, China.

4 Research Center of Avian Disease, College of Veterinary Medicine, Sichuan Agricultural University, Chengdu 611130, China

5 Key Laboratory of Microbial Pathogenesis and Interventions of Fujian Province University, the Key Laboratory of Innate Immune Biology of Fujian Province, Biomedical Research Center of South China, Key Laboratory of OptoElectronic Science and Technology for Medicine of the Ministry of Education, College of Life Sciences, Fujian Normal University, Fuzhou 350117, China

*Address correspondence to Songying Ouyang, [ouyangsy@fjnu.edu.cn](mailto:ouyangsy@fjnu.edu.cn), Mafeng Liu, 14049@sicau.edu.cn and Anchun Cheng, anchun@sicau.edu.cn.

*^#^* M.W., D.Z. and X.T. contributed equally to this work. Author order was determined by flipping a coin.

This file includes:

Supplementary Materials and Methods, Figures S1 to S5, Table S1 to S4

**Supplementary Materials and Methods**

**Media and growth conditions**

The parent strain *R. anatipestifer* CH-1 and mutant derivatives, *R. columbina* were grown in GCB medium [[1](#_ENREF_1)] or blood LB agar (LB agar supplemented with 5% defibrinated sheep blood) at 37°C in an aerobic chamber. F. columnare was grown in F. columnare growth medium (FCGM) or on FCGM agar at 28°C in an aerobic chamber. The FCGM composition per L includes 8 g tryptone, 0.8 g yeast extract, 1 g MgSO_4_·7H_2_O, 0.65 g CaCl_2_, 5 g NaCl, and 1.5 g sodium citrate tribasic dihydrate. *F. soli* was purchased from the China General Microbiological Culture Collection Center (CGMCC). According to the CGMCC guidelines, *F. soli* was grown in TSB medium or TSB agar at 28°C in an aerobic chamber. *E. coli* strains DH5α, S17-1, BL21(DE3) or Rosetta were grown in LB medium and incubated aerobically at 37°C. To restrict the free iron, iron chelator EDDHA (Alfa Chemistry, USA) or Dip (Sigma, China) was added to the medium. δ-Aminolevulinic acid was dissolved in double-distilled water and filter sterilized through a 0.22-μm Millipore filter (Millipore, China). Haemin (Sigma, USA) was dissolved immediately before use in 0.1 N NaOH and sterilized through a 0.22-μm Millipore filter for haemin binding experiment. Duck haemoglobin was prepared as previously described [[2](#_ENREF_2)], and sterilized with a 0.45-μm filter. Antibiotics were supplemented as follows: For *E. coli*, ampicillin (Amp) was used at 100 μg/mL. For *R. anatipestifer* CH-1, cefoxitin (Cfx) was used at 1 μg/mL and Spc was used at 80 μg/mL.

**Construction of the mutant and complementation strains**

Creation of the markerless deletion was performed as described previously [[3](#_ENREF_3)]. Briefly, a ~800-bp upstream fragment and a ~800-bp downstream fragment were PCR amplified from the chromosomal DNA of RA CH-1 and were joined using overlap extension (SOE) PCR. Concatenated fragments were then ligated into the suicide vector pOBS [[3](#_ENREF_3)]. Correct clones were selected using PCR and sequence verified. A verified clone was then introduced into *R. anatipestifer* CH-1 or its derivative strains via conjugation. Gene deletions were confirmed via PCR after counter-selection.

Gene-complementation plasmids were constructed using the plasmid pLMF03 as a skeleton as previously described [[4](#_ENREF_4)]. The complementation plasmid was introduced into the mutant strain by conjugation as described in a previous study [[5](#_ENREF_5)].

**Site-directed mutagenesis**

Single substitutions were introduced into *rhuH* using the natural transformation-based method as previously described [[1](#_ENREF_1)]. Briefly, taking the mutation of His96 an ALA as an example, an upstream fragment and a downstream fragment, which contains both mutant nucleotides, were amplified from chromosomal DNA of RA CH-1 by PCR, using primers RhuH A-upP1/RhuH 96^H-A^upP2 and RhuH 96^H-A^downP1/ RhuH A-downP2 (Table S2), and two fragments were joined using SOE PCR. The recombination fragment was introduced into recipient strain RA CH-1Δ*rhuH::cfx*-*sacB*, which was then spread onto an LB blood plate containing 15% sucrose. Correct clones were PCR selected and sequence verified.

**Plasmid construction for expression**

The *rhuH* gene without the signal peptide sequence (the first 57 bp) was PCR amplified from *R. anatipestifer* CH-1 genomic DNA using primers RhuH ExpP1 and RhuH ExpC-HisP2 (Table S2) and cloned into plasmid pET32a to create plasmid pET32a*::rhuH*. The *rhuH* gene lacking the signal peptide sequence was PCR amplified from the genomic DNA of *R. anatipestifer* CH-1 using primers RhuH GSTP1 and RhuH GSTP2 (Table S2), and the entire coding region of *hasA* was amplified by PCR from *S. marcescens* using primers HasA GSTP1 and HasA GSTP2 (Table S2). The *rhuH^RC^* gene lacking the signal peptide sequence (the first 42 bp) was PCR amplified from the genomic DNA of *R. columbina* using primers RhuH^RC^ GSTP1 and RhuH^RC^ GSTP2 (Table S2), the *rhuH^FC^* gene lacking the signal peptide sequence (the first 60 bp) was PCR amplified from the genomic DNA of *F. columnare* using primers RhuH^FC^ GSTP1 and RhuH^FC^ GSTP2 (Table S2), and the *rhuH^FS^* gene lacking the signal peptide sequence (the first 60 bp) was PCR amplified from the genomic DNA of *F. soli* using primers RhuH^FS^ GSTP1 and RhuH^FS^ GSTP2 (Table S2). Each PCR product was cloned into the plasmid pGEX-4T-1, which contains an N-terminal GST tag followed by a thrombin cleavage site, to create plasmids pGEX-4T-1*::rhuH*, pGEX-4T-1*::hasA*, pGEX-4T-1*::rhuH^RC^*, pGEX-4T-1*::rhuH^FC^* and pGEX-4T-1*::rhuH^FS^*.

For plasmid construction for site-directed mutagenesis, taking the mutation of the His96 to ALA as an example, *rhuH* with the 96^H-A^ point mutation was PCR amplified from *R. anatipestifer* CH-1 *rhuH^96H-A^* genomic DNA with primers RhuH ExpP1 and RhuH ExpC-HisP2 (Table S2). The amplified fragment was cloned into pET32a to give pET32a*::rhuH^96H-A^*.

**Production and purification of recombinant protein**

*E. coli* strain Rosetta was transformed with pET32a*::rhuH*, pBAD24*::hasA* and pBAD24*::recA*, and grown overnight in LB with Amp at 37°C. Cells were subcultured into LB medium with Amp and grown to the mid-logarithmic phase at 37°C before the addition of 0.5 mM isopropyl-1-thiol-D-galactopyranoside (IPTG). Culture was continued to grow for another 4 h at 37°C before harvesting. Cells were harvested and lysed via lysozyme and DNase I. Insoluble debris was removed via centrifugation, and the supernatant was mixed with His-binding Ni-NTA resin. The purified protein was washed twice with 50 mM Tris-HCl to remove any imidazole. 500 μL of eluate RhuH protein was loaded into Superdex 75 Increase 10/300 GL (Cytiva, Buckinghamshire, UK) column pre-equilibrated with buffer containing 100 mM Tris (pH 7.0) and 250 mM NaCl. The flow rate was 0.3 mL/min. Absorbance was recorded at 280 nm and 410 nm.

Competent *E. coli* BL21(DE3) was transformed with pGEX-4T-1*::rhuH*, pGEX-4T-1*::hasA*, pGEX-4T-1*::rhuH^RC^*, pGEX-4T-1*::rhuH^FC^* and pGEX-4T-1*::rhuH^FS^*, and grown overnight in LB plus Amp at 37°C. Cells were grown to the mid-logarithmic phase of growth at 37°C, induced with 0.5 mM IPTG, and grown for another 3 h. Cells were then lysed with lysozyme and DNase I. Insoluble debris was removed via centrifugation, and the supernatant was harvested. The recombinant protein was purified with glutathione-sepharose 4B (Cytiva, USA) according to the manufacturer’s instructions.

**Antibody preparation**

The antibody against rRhuH was prepared as previously described [[6](#_ENREF_6)]. The initial injection into 4-week-old Kunming mice was an equal mixture of rRhuH and Freund’s complete adjuvant (Sigma, China). After two weeks, rRhuH and Freund’s incomplete adjuvant (Sigma, China) were inoculated twice (at half-monthly intervals) into 4-week-old Kunming mice. Blood samples were taken every two weeks by retro-orbital bleeding and were centrifuged at 8,000× *g* for 10 min at 4°C. Sera were stored at −20°C.

**Growth experiments**

R. anatipestifer CH-1 and its derivative strains were cultured overnight in GCB liquid medium at 37°C with shaking and then inoculated into 20 mL of GCB or GCB supplemented with 120 μM EDDHA or 120 μM EDDHA and 0.3 μM duck haemoglobin (Hb)/0.5 μM haemin at OD_600_ of 0.1. The OD_600_ was monitored every 2 h up to 14 h. The assay was performed in three independent replicates.

**RT-qPCR**

RT-qPCR was performed as previously described [[4](#_ENREF_4)]. Briefly, *R. anatipestifer* CH-1 (WT), *R. anatipestifer* CH-1*Δfur* (*Δfur*), *R. anatipestifer* CH-1*Δfur* pLMF03::*fur* (*Δfur^C^*), and *R. anatipestifer* CH-1*^ΔFur box^* (WT*^ΔFur box^*) strains were grown in GCB liquid medium; the WT strain was grown in GCB or GCB treated with 120 μM EDDHA to the exponential phase [OD] at 600 nm = 1–1.5. Total RNA was extracted using the RNeasy Minikit procedure (Qiagen), and cDNA was synthesized using reverse transcriptase (Vazyme, China). RT-qPCR was performed using the SYBR Green master mix (Vazyme) using the primer pairs in Table S2. Amplification was achieved using a CFX Connect real-time PCR detection system. Relative fold changes were calculated using the threshold cycle (ΔΔCT) method normalized by the *R. anatipestifer* housekeeping 16s rRNA gene.

**Cell fractionations and protein localization**

Membrane proteins were prepared as previously described [[7](#_ENREF_7)]. Cells were resuspended in 20 mM Tris-HCl (pH 7.4), 10 mM EDTA, 1 mM *N-α-p*-tosyl-_L_-lysine chloromethyl ketone (TLCK), lysed with a French-press cell disrupter (Thermo Scientific, USA), and then centrifuged at 8,000× *g* for 30 min at 4°C. The sediment was resuspended in TLCK and stored at −80°C.

Secreted proteins were prepared as previously described [[6](#_ENREF_6)]. Briefly, bacteria were grown overnight at 37°C with shaking in GCB or GCB containing 100 μM Dip. Samples were harvested at the stationary phase [OD] at 600 nm = 3 and centrifuged for 10,000× *g* for 10 min at 4°C. Inactive secreted proteins were concentrated by precipitation with 10% trichloroacetic acid as described previously [[6](#_ENREF_6)] and were centrifuged at 12,000× *g* for 20 min at 4°C. The pellet was resuspended in a protein-loading buffer and stored at −20°C.

**Western blotting**

Cell fractionations were separated by SDS-PAGE, and electroblotted onto nitrocellulose membranes, which were then blocked with 5% skim milk in TBST overnight. The polyclonal antibody against RhuH or RhuA was used to probe the membranes at 1:400 dilution in TBST buffer for 3 h, followed by a 1:2000 dilution of horseradish Peroxidase (HRP)-conjugated AffiniPure goat anti-mouse IgG (Proteintech, China) for 1 h. Signals were detected using ECL reagents (Bio-Rad, USA) in a ChemiDoc MP imaging system (Bio-Rad, USA).

**Extraction of outer membrane vesicles (OMVs)**

After culturing *R. anatipestifer* CH-1 in GCB or GCB medium supplemented with 100 μM Dip until reaching the stationary phase, the supernatant was collected by centrifuging at 7,000 rpm for 20 min. The collected supernatant was filtered using a 0.45-μm filter. The filtrate was then centrifuged at 100,000× *g* for 2 h at 4 °C to concentrate OMVs into a 1-mL volume following the removal of the remaining supernatant.

**Transmission electron microscopy (TEM) detection**

*R. anatipestifer* CH-1 was grown in TSB to the exponential phase (OD600 of approximately 1–1.5). Bacteria (10^9^ CFUs) were harvested and washed three times with PBS. Bacteria were then resuspended in 3% glutaraldehyde for 2 h and then fixed using 1% osmium tetroxide. Samples were dehydrated using a gradient of acetone (30%, 50%, 70%, 80%, 90%, 95%, and 100%) and then embedded in epoxy resin (Ep812, SPI). Ultrathin sections of approximately 60–90 nm thickness were prepared using an ultramicrotome (UC7rt, LEICA), mounted onto copper grids, and stained with 0.5% uranyl acetate at room temperature for 10–15 min, followed by 0.5% lead citrate staining at room temperature for 1–2 min. Samples were imaged using TEM (JEM-1400FLASH, JEOL, Japan) operating at 80 kV to observe specific changes. For TEM imaging, OMVs were dropped onto the copper grid of the support membrane for 5 min. Excess liquid was removed, and samples were stained with 1% phosphotungstic acid for 1–2 min. OMVs were then observed via TEM at 80 kV.

**Absorption spectroscopy**

Increasing amounts of haemin (0–40 μM) were added into a cuvette containing 20 μM recombinant RhuH in 1 mL of 50 mM Tris (pH 8.0) and 100 mM NaCl. As a control, the same amount of haemin was added to 1 mL of buffer alone. Samples were incubated for 5 min at 37°C, followed by spectrophotometry (300–700 nm) using a Nanodrop 2000. The binding ratio of haemin to rRhuH was determined by plotting the change in absorbance at 407 nm between the control and the rRhuH sample.

**Virulence and colonization assay**

Virulence and colonization assays were performed using previously described methods [[6](#_ENREF_6)]. For the virulence assay, 3-day-old ducklings ((10 per group) were inoculated in the leg with 10^9^ CFU of RA CH-1(WT), *ΔrhuH* or *rhuH^96H-A^* in 200 μL of PBS and monitored for near mortality. For the colonization assay, 3-day-old ducklings (6 per group) were similarly inoculated with 10⁹ CFU of the respective strains in 200 μL of PBS. After 24 h inoculation, ducklings were euthanized by forced inhalation of CO₂. Heart blood, liver, and spleen tissues were collected, weighed, homogenized, and diluted in PBS. The dilutions were then plated on blood agar to determine the number of CFUs of bacteria per ml gram of tissues. The results were presented graphically as the CFU per gram of tissue at 24 h postinfection.

**Ethics statement**

This study was conducted in accordance with the good animal experimental practice standards of the local animal welfare institution and the ethics committee of Sichuan Agricultural University (20240327). All procedures involving animals were reviewed and approved by the Ethics Committee of Sichuan Agricultural University, and the study was conducted in accordance with the ARRIVE guidelines.

**Bioinformatic assays**

The RhuH and its homologous sequences were obtained from the NCBI database. Sequences were aligned using ClustalW [[8](#_ENREF_8)], and the phylogenetic tree was constructed using the neighbor-joining method (bootstrap replicates×1,000) by MEGA7 software. Phylogenetic tree was edited using iTOL v6 (<https://itol.embl.de>). Alphafold3 (<https://golgi.sandbox.google.com/>) [[9](#_ENREF_9)] was employed to predict the complex structure of RhuH-haem, as well as the structures of the RhuH^RC^, RhuH^FC^ and RhuH^FS^ proteins, and these structures were visualized using PyMOL software.

**Figure S1.** **Size exclusion chromatography of recombinant RhuH protien purification**

**
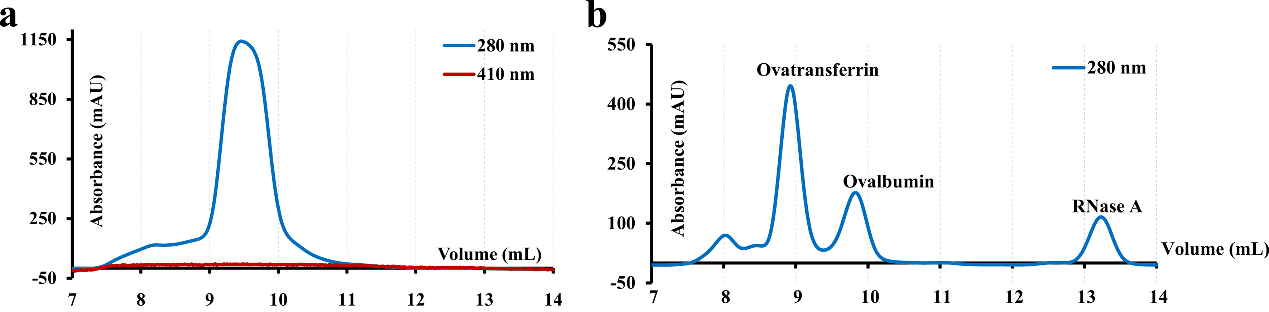
**

**Figure S1. Size exclusion chromatography of recombinant RhuH protien purification. (a)** The RhuH protein was loaded onto a Superdex 75 column, equilibrated with a buffer containing 100 mM Tris (pH 7.0) and 250 mM NaCl, and chromatographed at a flow rate of 0.3 mL/min. **(b)** The calibration profile of ovatransferrin (76 kDa), ovalbumin (44.5 kDa), and RNase A (13.7 kDa).

**Figure S2 Comparison of three-dimensional protein structures of six haemophores**

**
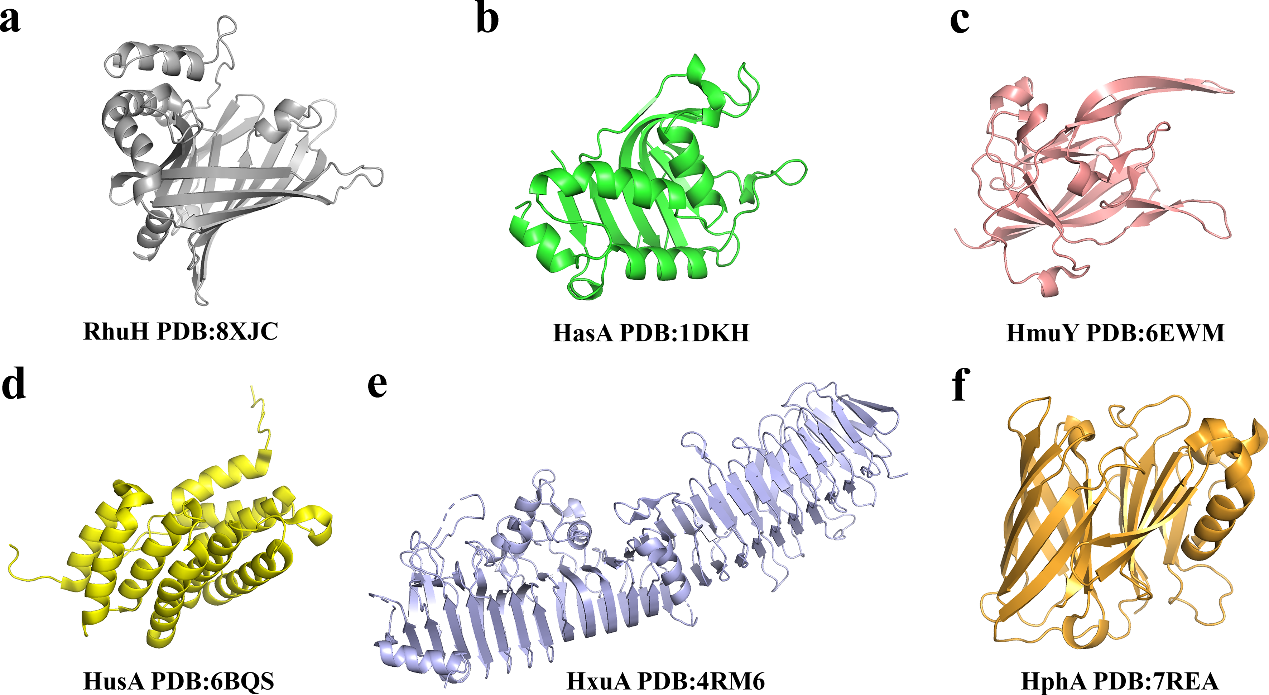
**

**Figure S2. Comparison of three-dimensional protein structures of six haemophores.** (a) RhuH (PDB:8XJC) in *R. anatipestifer*; (b) HasA (PDB:1DKH) in *S. marcescens* [[10](#_ENREF_10)], (c) HmuY (PDB:6EWM) [[11](#_ENREF_11)] in *P. gingivalis*; (d) HusA (PDB:6BQS) [[12](#_ENREF_12)] in *P. gingivalis*; (e) HxuA (PDB: 4RM6) in *H. influenzae* [[13](#_ENREF_13)]; (f) HphA (PDB:7REA) in *Acinetobacter baumannii* [[14](#_ENREF_14)]. Three-dimensional protein structures visualized using pymol.

**Figure S3.** **Glutamic acid residue Glu160 is a key residue for dimer formation**

**
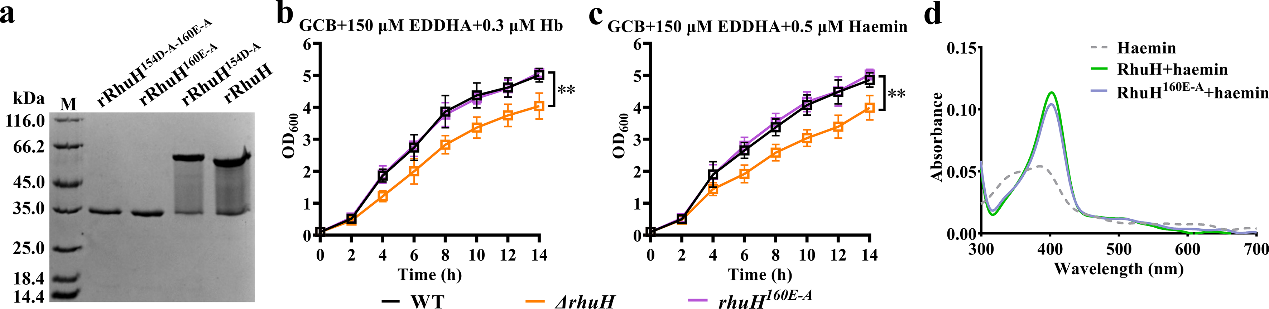
**

**Figure S3. Glutamic acid residue GLU-160 is a key residue for dimer formation. (a)** The SDS-PAGE gel shows the separation of recombinant proteins rRhuH^154D-A-160E-A^, rRhuH^160E-A^, rRhuH^154D-A^, and rRhuH. M: molecular weight. **(b** and **c)** Growth curves of WT, *ΔrhuH*, and RA CH-1 *rhuH^160E-A^* (*rhuH^160E-A^*) strains under iron-limited GCB supplemented with 0.3 µM duck Hb (b) or 0.5 µM haemin (c). Data represents means plus standard deviations from three independent experiments each in biological triplicate. **(d)** Absorption spectra of 20 μM RhuH or RhuH^160E-A^ binding to 20 μM haemin.

**Figure S4. Comparative analysis of the RhuH crystal structure and AlphaFold3-predicted structure**

**
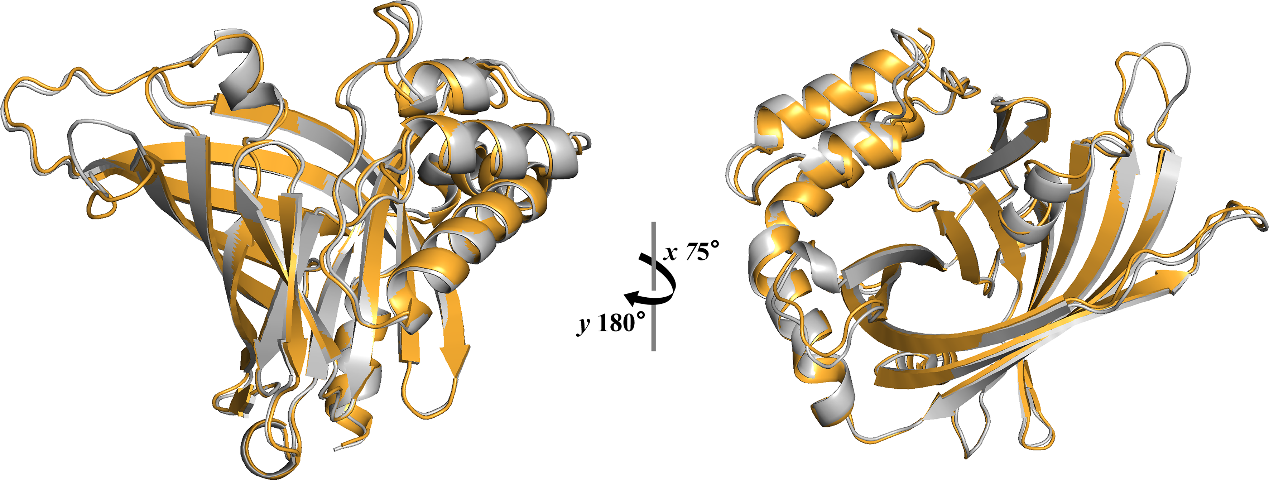
**

**Figure S4. Comparative analysis of the RhuH crystal structure and AlphaFold3-predicted structure.** The 3D model of RhuH constructed using AlphaFold3 is shown in orange. This model was compared to the crystal structure of RhuH (PDB ID: 8XJC), represented in gray. The overall structures were largely similar, with only minor differences in some loops.

**Figure S5. Virulence and colonization efficiency of *R. anatipestifer* CH-1, *ΔrhuH* and *rhuH^96H-A^* in ducklings infection model**

**
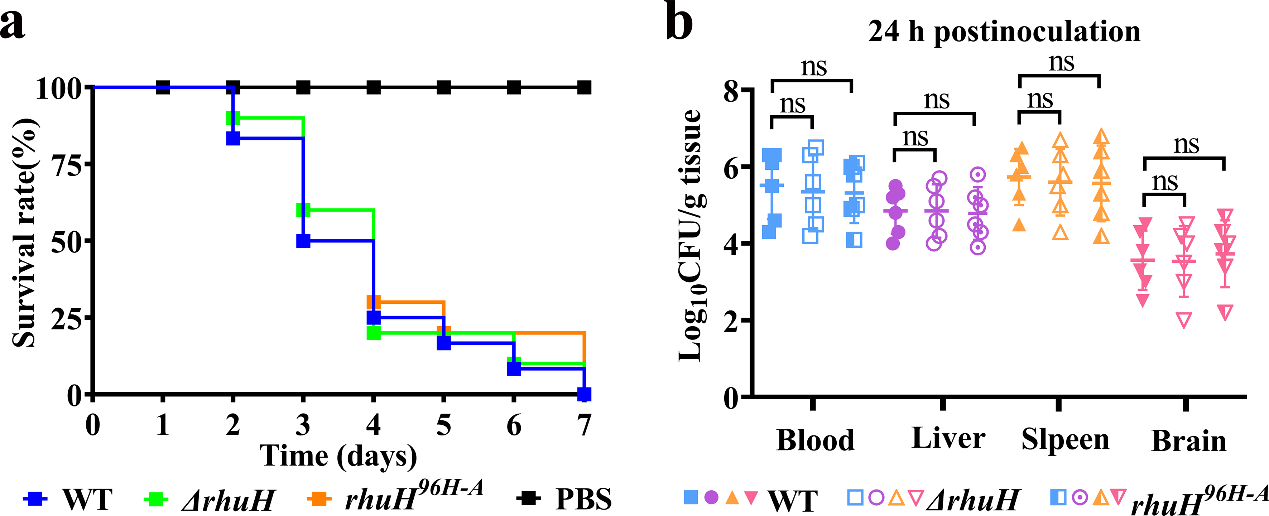
**

**Figure S5. Virulence and colonization efficiency of *R. anatipestifer* CH-1, *ΔrhuH* and *rhuH^96H-A^* in ducklings infection model. (a)** Ten 3-day-old ducklings were inoculated with 10^9^ CFU of each strain, while PBS injection served as a negative control. The survival of the ducklings was monitored over a 7-day period. Survival rates were displayed as Kaplan–Meier survival curves using GraphPad Prism 9 software. **(b)** Colonization of each strain in ducklings at 24 h post-infection. Six 3-day-old ducklings were inoculated with 10^9^ CFU of each strain, and bacterial loads were recovered from heart blood, liver, spleen, and brain 24 h after infection. The horizontal black bars represent the standard deviations (SDs). Statistical analyses were conducted using unpaired t-test. ns: not significant.

**Table S1. Strains and plasmids used in this study**

| ***E.coli* strains** | **Genotype** | | **Source or reference** |
| --- | --- | --- | --- |
| DH5α | | F^-^*,φ80dlacZ*Δ*M15,*Δ(*lacZYA-argF*)*U169, deoR, recA1, endA1*, *hsdR17 (rK^-^, mK^+^), phoA, supE44, λ^-^, thi-1, gyrA96*, *relA1* | Laboratory collection |
| S17-1 | | *hsdR17 recA1* RP4-2-tet::Mu-1kan::Tn7; Sm^R^ | [[15](#_ENREF_15)] |
| Rosetta | | *F-, ompT hsdSB (rB-mB-) gal dcm (DE3) pRARE2 (CamR)* | Laboratory collection |
| JP313 | | *araD139 relA rpsL 150 thi flb5301 (lacU 139) deo7 ptsF25*  *∆ara 174* | Laboratory collection |
| BL21(DE3) | | *E. coli* B F^-^ *dcm ompT hsdS(rB^-^ mB^-^) gal lon* λ(DE3 [*lacI lacUV5-T7* gene *1 ind1 sam7 nin5*]) | Laboratory collection |
| BL21(DE3) pGEX-4T-1::*rhuH* | | BL21(DE3), pGEX-4T-1*::rhuH*, Amp^R^ | This study |
| BL21(DE3) pGEX-4T-1::*hasA* | | BL21(DE3), pGEX-4T-1*::hasA*, Amp^R^ | This study |
| Rosetta pET32a*::rhuH* | | Rosetta, pET32a*::rhuH*, Amp^R^ | This study |
| JP313 pBAD24*::recA* | | Rosetta, pET32a*::recA*, Amp^R^ | [[6](#_ENREF_6)] |
| JP313 pBAD24*::hasA* | | Rosetta, pET32a*::hasA*, Amp^R^ | [[6](#_ENREF_6)] |
| JP313 pBAD24*::rhuA* | | JP313, pBAD24*::rhuA,* Amp^R^ | [[6](#_ENREF_6)] |
| C600*ΔhemA* | | C600 (F- *thr leu lacY thi supE* Δ*hemA*::Km), Km^R^ | [[16](#_ENREF_16)] |
| C600*ΔhemA* pAM238*::hemR* | | C600*∆hemA*, pAM238*::hemR*, Cfx^R^ | [[6](#_ENREF_6)] |
| Rosetta pET32a*::rhuH^154D-A-160E-A^* | | Rosetta, pET32a*::rhuH^154D-A-160E-A^*, Amp^R^ | This study |
| Rosetta pET32a*::rhuH^154D-A^* | | Rosetta, pET32a*::rhuH^154D-A^*, Amp^R^ | This study |
| Rosetta pET32a*::rhuH^160E-A^* | | Rosetta, pET32a*::rhuH^160E-A^*, Amp^R^ | This study |
| Rosetta pET32a*::rhuH^96H-A^* | | Rosetta, pET32a*::rhuH^96H-A^*, Amp^R^ | This study |
| BL21(DE3) pGEX-4T-1::*rhuH^RC^* | | BL21(DE3), pGEX-4T-1*::rhuH^RC^*, Amp^R^ | This study |
| BL21(DE3) pGEX-4T-1::*rhuH^FC^* | | BL21(DE3), pGEX-4T-1*::rhuH^FC^*, Amp^R^ | This study |
| BL21(DE3) pGEX-4T-1::*rhuH^FS^* | | BL21(DE3), pGEX-4T-1*::rhuH^FS^*, Amp^R^ | This study |
| **Strains** | | **Phenotype or genotype** | **Source or reference** |
| *Riemerella anatipestifer* CH-1 (RA CH-1) | | *R. anatipestifer serotype* 1, Km^R^, Erm^R^ | [[17](#_ENREF_17)] |
| RA CH-1*ΔrhuH* | | RA CH-1, *rhuH* mutant | This study |
| RA CH-1 pLMF03 | | RA CH-1, pLMF03, Cfx^R^ | This study |
| RA CH-1*ΔrhuH* pLMF03 | | RA CH-1, *rhuH* mutant, pLMF03, Cfx^R^ | This study |
| RA CH-1*ΔrhuH* pLMF03*::rhuH* | | RA CH-1, *rhuH* mutant, pLMF03*::rhuH*, Cfx^R^ | This study |
| RA CH-1 *rhuH^154D-A^* | | RA CH-1, *rhuH* point mutant, 154^D-A^ | This study |
| RA CH-1 *rhuH^160E-A^* | | RA CH-1, *rhuH* point mutant, 160^E-A^ | This study |
| RA CH-1 *rhuH^154D-A-160E-A^* | | RA CH-1, *rhuH* point mutant, 154^D-A^160^E-A^ | This study |
| RA CH-1 *rhuH^96H-A^* | | RA CH-1, *rhuH* point mutant, 96^H-A^ | This study |
| RA CH-1 WT*^ΔFur box^* | | RA CH-1, Fur box mutant in the promoter region of *rhuH*, Spc^R^ | This study |
| *Riemerella columbina* | | *R. columbina* CCUG 47689 | [[18](#_ENREF_18)] |
| *Flavobacterium columnare* | | *F. columnare* | This study |
| *Flavobacterium soli* | | *F. soli* DSM 19725 | CGMCC |
| **Plasmids** | | **Genotype** | **Source or reference** |
| pLMF03 | | Shuttle plasmid, *ermF* promoter, *ori*ColE1, *ori*pRA0726, Amp^R^, Cfx^R^ | [[4](#_ENREF_4)] |
| pLMF03*::rhuH* | | pLMF03 carrying *rhuH* from RA CH-1, Amp^R^, Cfx^R^ | This study |
| pET32a | | T7 promoter, His-tag, Amp^R^ | This study |
| pET32a*::rhuH* | | pET32a carrying *rhuH* from RA CH-1, Amp^R^ | This study |
| pET32a*::hasA* | | pET32a carrying *hasA* from *S. marcescens*, Amp^R^ | This study |
| pET32a*::recA* | | pET32a carrying *recA* from RA CH-1, Amp^R^ | [[4](#_ENREF_4)] |
| pBAD24*::**rhuA* | | pBAD24 carrying *rhuA* from RA CH-1, Amp^R^ | [[6](#_ENREF_6)] |
| pAM238 | | pSC101 origin, Spc^R^ | [[19](#_ENREF_19)] |
| pAM238*::hemR* | | pAM238 carrying *hemR* from *S. marcescens*, Spc^R^ | This study |
| pOBS | | Suicide plasmid, *sacB*^+^, Amp^R^, Cfx^R^ | [[3](#_ENREF_3)] |
| pOBS*ΔrhuH* | | pOBS carrying *rhuH* upstream and *rhuH* downstream from RA CH-1, Amp^R^, Cfx^R^ | This study |
| pGEX-4T-1 | | Tac promoter, GST-tag, Amp^R^ | Laboratory collection |
| pGEX-4T-1*::rhuH* | | pGEX-4T-1 carrying *rhuH* from RA CH-1,Amp^R^ | This study |
| pGEX-4T-1*::hasA* | | pGEX-4T-1 carrying *hasA* from RA CH-1,Amp^R^ | This study |
| pET32a*::rhuH**^154D-A-160E-A^* | | pET32a carrying 154^D-A^160^E-A^ mutant of RA CH-1, Amp^R^ | This study |
| pET32a*::rhuH^154D-A^* | | pET32a carrying 154^D-A^ mutant of RA CH-1, Amp^R^ | This study |
| pET32a*::rhuH^160E-A^* | | pET32a carrying 160^E-A^ mutant of RA CH-1, Amp^R^ | This study |
| pET32a*::rhuH^96H-A^* | | pET32a carrying *rhuH* 96^H-A^ mutant of RA CH-1, Amp^R^ | This study |
| pGEX-4T-1*::rhuH^RC^* | | pGEX-4T-1 carrying *rhuH_RC_* from *R. columbina*, Amp^R^ | This study |
| pGEX-4T-1*::rhuH^FC^* | | pGEX-4T-1 carrying *rhuH^FC^* from *F. columnare*, Amp^R^ | This study |
| pGEX-4T-1*::rhuH^FS^* | | pGEX-4T-1 carrying *rhuH^FS^* from *F. soli*, Amp^R^ | This study |

Amp^R^, ampicillin resistance; Spc^R^, spectinomycin resistance; Cfx^R^, cefoxitin resistance. Km^R^, kanamycin resistance. Erm^R^, erythromycin resistance.

**Table S2. Primers used in this study**

| **Primers** | **Organism** | **Sequence ( 5'－3')** |
| --- | --- | --- |
| Cfx P1 | pLMF03 | GGTGCTGCAATGTTGATG |
| Cfx P2 | pLMF03 | CCGCTAAGGTATAACTG |
| 16S qRTP1 | RA CH-1 | ATGCGAAAGGAGGATTGC |
| 16S qRTP2 | RA CH-1 | TTACACCTCAAATACCTC |
| RhuH upP1 | RA CH-1 | CCGCTCGAGGTGTCATTTTTAAAACCAG |
| RhuH upP2 | RA CH-1 | CAACGATATGATTACTTTAATTATTTTC |
| RhuH downP1 | RA CH-1 | GAAAATAATTAAAGTAATCATATCGTTG |
| RhuH downP2 | RA CH-1 | GGACTAGTCGTATGAAATTAGCAGCTATAG |
| RhuH CompP1 | RA CH-1 | CATGCCATGGATGAAATCAAAGACATTACTC |
| RhuH CompP2 | RA CH-1 | GACTAGTTTATTTAACGATGTATTTTTTTAC |
| rHasA P1 | *S.marcescens* | ACGCGTCGACGTCGGCCATAGCGGATGGCATTTTCAGTCAATTATGACAGCAGC |
| rHasA P2 | *S.marcescens* | ACATGCATGCATGTTCAGTGGTGGTGGTGGTGGTGGGCCGCCAGCAGTTCC |
| RhuH ExpupP1 | RA CH-1 | CCGGAATTCCATATGCAAGATAAAAGAGATATTACAGCC |
| RhuH ExpC-HisP2 | RA CH-1 | CCCAAGCTTGGGTTAGTGGTGGTGGTGGTGGTGTTTAACGATGTATTTTTTTACC |
| RhuH GSTP1 | RA CH-1 | CCGGAATTCCAAGATAAAAGAGATATTACAGCC |
| RhuH GSTP2 | RA CH-1 | CCGCTCGAGTTATTTAACGATGTATTTTTTTAC |
| HasA GSTP1 | *S.marcescens* | CCGGAATTCGCATTTTCAGTCAATTATGAC |
| HasA GSTP2 | *S.marcescens* | CCGCTCGAGTCAGGCCGCCAGCAGTTCCGG |
| RhuH qRTP1 | RA CH-1 | CAGGTTAAAGGACAATGGACAC |
| RhuH qRTP2 | RA CH-1 | ATATTCTCTTCTAGGGAGCGG |
| SacB P1 | pEX18GM | CTAGTCTAGACTAGTTTTTTTTAACATTTGATTTTGTAT |
| SacB P1 | pEX18GM | ACGCGTCGACGTCGGTTATTTGTTAACTGTTAATTGTCCT |
| RhuH A-upP1 | RA CH-1 | CCGCTCGAGTTTTAATATCATTAACCGT |
| RhuH A-downP2 | RA CH-1 | GACTAGTCCTGTTGCTGATGGGCTTATAC |
| RhuH 154^D-A^upP2 | RA CH-1 | CCAAAATGTTTTTTCTGCTAAGTGTATCCAG |
| RhuH 154^D-A^downP1 | RA CH-1 | CTGGATACACTTAGCAGAAAAAACATTTTGG |
| RhuH 160^E-A^upP2 | RA CH-1 | CATCTGCAGTACTTGCCCAAAATGTT |
| RhuH 160^E-A^downP1 | RA CH-1 | AACATTTTGGGCAAGTACTGCAGATG |
| RhuH 154^D-A^160^E-A^upP2 | RA CH-1 | CAGTACTTGCCCAAAATGTTTTTTCTGCTAAGTGTATC |
| RhuH 154^D-A^160^E-A^downP1 | RA CH-1 | GATACACTTAGCAGAAAAAACATTTTGGGCAAGTACTG |
| RhuH 96^H-A^upP2 | RA CH-1 | CTTGTCTCCATGCTTTTACAATAGCATTTT |
| RhuH 96^H-A^downP1 | RA CH-1 | ATTGTAAAAGCATGGAGACAAGATTGGC |
| Fur box upP1 | RA CH-1 | GCATTTCTTTCTTATTTGTATAACGAGAAGCC |
| Fur box upP2 | RA CH-1 | TATAACATGTATTCACGAAAAAAACTGATATTTATCATT |
| Fur box spcP1 | pAM238 | AATGATAAATATCAGTTTTTTTCGTGAATACATGTTATA |
| Fur box spcP2 | pAM238 | TCTTTGCAAATCTGCTTACCAATTAGAATG |
| Fur box downP1 | RA CH-1 | TCTAATTGGTAAGCAGATTTGCAAAGAAAA |
| Fur box downP2 | RA CH-1 | CTGGGCAGATATTTCTAAAATATTTGTCCC |
| RhuH^FC^ GSTP1 | *F. columnare* | GGTTCCGCGTGGATCCAACAAAAAAGAAGAAGATAGAAAAGCG |
| RhuH^FC^ GSTP2 | *F. columnare* | GCTGCATGTGTCAGAGGTTTTATTTACTAATATATGTGTC |
| RhuH^RC^ GSTP1 | *R. columbina* | CCGGAATTCTGCCTAATGAAGGCTCAAAAAC |
| RhuH^RC^ GSTP2 | *R. columbina* | CCGCTCGAGTTATTTTTCAATATATTCATTC |
| RhuH^FS^ GSTP1 | *F. soli* | CTGGTTCCGCGTGGATCCGACAAGAAAAAAGAAGACATCAAAGCC |
| RhuH^FS^ GSTP2 | *F. soli* | GCTGCATGTGTCAGAGGTTTTATTTTCTTACAAAGCTGTC |

**Table S3. X‐ray crystallography data collection and refinement statistics**

| **Dataset** | **RhuH (8XJC)** |
| --- | --- |
| Beamline | BL-10U02 |
| Wavelength (Å) | 0.979183 |
| Space group | *P*2_1_2_1_2 |
| Unit Cell |  |
| a, b, c (Å) | 110.48, 1120.06, 53.85 |
| α, β, γ (◦) | 90.00, 90.00, 90.00 |
| Resolution (Å) | 60.03-2.85 (2.95 -2.85) |
| Unique reflections | 17335 (1569) |
| R-merge | 0.093 (0.3463) |
| *Mean I/sigma(I)* | 7.63 (1.98) |
| CC_1/2_ | 0.971 (0.728) |
| R-meas | 0.1318 (0.4898) |
| Completeness (%) | 0.97 (1.00) |
| Multiplicity | 11.2 (8.9) |
| Reflections used in refinement | 16827 (1569) |
| Reflections used for R-free | 1691 (159) |
| R-work | 0.2247 (0.2971) |
| R-free | 0.2507 (0.3331) |
| Wilson B-factor (Å^2^) | 46.09 |
| Number of non-hydrogen atoms |  |
| Macromolecules | 4709 |
| Protein residues | 555 |
| RMS(bonds) | 0.016 |
| RMS(angles) | 1.66 |
| Ramachandran favored (%) | 97.0 |
| Ramachandran allowed (%) | 3.0 |
| Ramachandran Outliers (%) | 0 |
| Rotamer outliers (%) | 0.79 |
| Clashscore | 3.01 |
| Average B-factor (Å^2^) | 46.90 |

Statistics for the highest-resolution shell are shown in parentheses.

**Table S4. GenBank accession numbers of RhuH and its homologous proteins in the order *Flavobacteriales*.**

| **GenBank accession numbers** | **Species** | **Family** |
| --- | --- | --- |
| WP_014938337.1 | *Riemerella anatipestifer* | *Weeksellaceae* |
| WP_002664234.1 | *Bergeyella zoohelcum* | *Weeksellaceae* |
| WP_018675666.1 | *Riemerella columbina* | *Weeksellaceae* |
| WP_031504050.1 | *Kaistella haifensis* | *Weeksellaceae* |
| WP_073180926.1 | *Cruoricaptor ignavus* | *Weeksellaceae* |
| WP_092735925.1 | *Riemerella columbipharyngis* | *Weeksellaceae* |
| WP_105246265.1 | *Apibacter adventoris* | *Weeksellaceae* |
| WP_233027928.1 | *Sinomicrobium kalidii* | *Flavobacteriaceae* |
| WP_123216407.1 | *Sinomicrobium pectinilyticum* | *Flavobacteriaceae* |
| WP_026706274.1 | *Flavobacterium soli* | *Flavobacteriaceae* |
| WP_077225713.1 | *Flavobacterium columnare* | *Flavobacteriaceae* |
| WP_091473244.1 | *Flavobacterium swingsii* | *Flavobacteriaceae* |
| WP_094157353.1 | *Flavobacterium psychrophilum* | *Flavobacteriaceae* |
| WP_187964761.1 | *Sinomicrobium weinanense* | *Flavobacteriaceae* |
| WP_140988967.1 | *Paucihalobacter ruber* | *Flavobacteriaceae* |
| WP_095072587.1 | *Tenacibaculum jejuense* | *Flavobacteriaceae* |
| WP_111363238.1 | *Flavobacterium tistrianum* | *Flavobacteriaceae* |
| WP_111379951.1 | *Flavobacterium nitrogenifigens* | *Flavobacteriaceae* |
| WP_132795598.1 | *Tenacibaculum skagerrakense* | *Flavobacteriaceae* |
| WP_268223442.1 | *Sinomicrobium oceani* | *Flavobacteriaceae* |
| WP_202005851.1 | *Flavobacterium tagetis* | *Flavobacteriaceae* |
| WP_072782951.1 | *Flavobacterium haoranii* | *Flavobacteriaceae* |
| WP_140998430.1 | *Flavobacterium profundi* | *Flavobacteriaceae* |
| WP_187657249.1 | *Flavobacterium macrobrachii* | *Flavobacteriaceae* |
| WP_093023040.1 | *Pustulibacterium marinum* | *Flavobacteriaceae* |
| WP_223679888.1 | *Flavobacterium hibisci* | *Flavobacteriaceae* |
| WP_119792948.1 | *Flavobacterium anhuiense* | *Flavobacteriaceae* |
| WP_166129122.1 | *Flavobacterium bernardetii* | *Flavobacteriaceae* |
| WP_116762423.1 | *Flavobacterium laiguense* | *Flavobacteriaceae* |
| WP_207294772.1 | *Flavobacterium endoglycinae* | *Flavobacteriaceae* |
| WP_110305687.1 | *Flavobacterium cheongpyeongense* | *Flavobacteriaceae* |
| WP_136465809.1 | *Flagellimonas onchidii* | *Flavobacteriaceae* |
| WP_203057637.1 | *Robertkochia sediminum* | *Flavobacteriaceae* |
| WP_188606322.1 | *Aquaticitalea lipolytica* | *Flavobacteriaceae* |
| WP_148543200.1 | *Seonamhaeicola marinus* | *Flavobacteriaceae* |
| WP_276473216.1 | *Maribacter huludaoensis* | *Flavobacteriaceae* |
| WP_111308948.1 | *Confluentibacter sediminis* | *Flavobacteriaceae* |
| WP_255841625.1 | *Abyssalbus ytuae* | *Flavobacteriaceae* |
| WP_308993657.1 | *Mariniflexile litorale* | *Flavobacteriaceae* |
| WP_121050331.1 | *Lacinutrix venerupis* | *Flavobacteriaceae* |
| WP_276390094.1 | *Eudoraea chungangensis* | *Flavobacteriaceae* |
| WP_132702570.1 | *Winogradskyella wandonensis* | *Flavobacteriaceae* |
| WP_027127548.1 | *Gelidibacter mesophilus* | *Flavobacteriaceae* |
| WP_281755251.1 | *Neptunitalea chrysea* | *Flavobacteriaceae* |
| WP_349241573.1 | *Asprobacillus argos* | *Flavobacteriaceae* |
| WP_242938152.1 | *Zhouia spongiae* | *Flavobacteriaceae* |
| WP_277898177.1 | *Galbibacter pacificus* | *Flavobacteriaceae* |
| WP_070236325.1 | *Urechidicola croceus* | *Flavobacteriaceae* |
| WP_163407723.1 | *Flavobacterium ajazii* | *Flavobacteriaceae* |
| [WP_070138615.1](https://www.ncbi.nlm.nih.gov/protein/WP_070138615.1?report=genbank&log$=prottop&blast_rank=10&RID=B90CHMAR016) | *Crocinitomix algicola* | *Crocinitomicaceae* |
| [WP_101333845.1](https://www.ncbi.nlm.nih.gov/protein/WP_101333845.1?report=genbank&log$=prottop&blast_rank=15&RID=B90CHMAR016) | *Brumimicrobium salinarum* | *Crocinitomicaceae* |
| WP_091511623.1 | *Flexibacter flexilis* | *Flexibacteraceae* |
| WP_073121336.1 | *Reichenbachiella agariperforans* | *Reichenbachiellaceae* |
| WP_124397859.1 | *Thermaurantimonas aggregans* | *Schleiferiaceae* |

Reference

[1] M. Liu, L. Zhang, L. Huang, F. Biville, D. Zhu, M. Wang, R. Jia, S. Chen, K. Sun, Q. Yang, Y. Wu, X. Chen, A. Cheng, *Appl Environ Microbiol* **2017**, *83* (9),

[2] M. Liu, M. Huang, L. Huang, F. Biville, D. Zhu, M. Wang, R. Jia, S. Chen, X. Zhao, Q. Yang, Y. Wu, S. Zhang, J. Huang, B. Tian, X. Chen, Y. Liu, L. Zhang, Y. Yu, L. Pan, M. Ur Rehman, A. Cheng, *Infect Immun* **2019**, *87* (8), e00072.

[3] X. Tian, L. Huang, M. Wang, F. Biville, D. Zhu, R. Jia, S. Chen, X. Zhao, Q. Yang, Y. Wu, S. Zhang, J. Huang, L. Zhang, Y. Yu, A. Cheng, M. Liu, *Vet Microbiol* **2020**, *247*, 108730.

[4] M. Liu, M. Wang, D. Zhu, M. Wang, R. Jia, S. Chen, K. Sun, Q. Yang, Y. Wu, X. Chen, F. Biville, A. Cheng, *Sci Rep* **2016**, *6*, 37159.

[5] M. Liu, Y. Huang, J. Liu, F. Biville, D. Zhu, M. Wang, R. Jia, S. Chen, X. Zhao, Q. Yang, Y. Wu, S. Zhang, X. Chen, Y. Liu, L. Zhang, Y. You, Y. Yu, A. Cheng, *Appl Microbiol Biotechnol* **2018**, *102* (17), 7475.

[6] M. Liu, S. Liu, M. Huang, Y. Wang, M. Wang, X. Tian, L. Li, Z. Yang, M. Wang, D. Zhu, R. Jia, S. Chen, X. Zhao, Q. Yang, Y. Wu, S. Zhang, J. Huang, X. Ou, S. Mao, Q. Gao, D. Sun, Y. L. Yu, A. Cheng, *Appl Environ Microbiol* **2021**, *87* (15), e0036721.

[7] J. K. Boonjakuakul, H. L. Gerns, Y. T. Chen, L. D. Hicks, M. F. Minnick, S. E. Dixon, S. C. Hall, J. E. Koehler, *Infect Immun* **2007**, *75* (5), 2548.

[8] M. A. Larkin, G. Blackshields, N. P. Brown, R. Chenna, P. A. McGettigan, H. McWilliam, F. Valentin, I. M. Wallace, A. Wilm, R. Lopez, J. D. Thompson, T. J. Gibson, D. G. Higgins, *Bioinformatics* **2007**, *23* (21), 2947.

[9] J. Abramson, J. Adler, J. Dunger, R. Evans, T. Green, A. Pritzel, O. Ronneberger, L. Willmore, A. J. Ballard, J. Bambrick, S. W. Bodenstein, D. A. Evans, C. C. Hung, M. O'Neill, D. Reiman, K. Tunyasuvunakool, Z. Wu, A. Žemgulytė, E. Arvaniti, C. Beattie, O. Bertolli, A. Bridgland, A. Cherepanov, M. Congreve, A. I. Cowen-Rivers, A. Cowie, M. Figurnov, F. B. Fuchs, H. Gladman, R. Jain, Y. A. Khan, C. M. R. Low, K. Perlin, A. Potapenko, P. Savy, S. Singh, A. Stecula, A. Thillaisundaram, C. Tong, S. Yakneen, E. D. Zhong, M. Zielinski, A. Žídek, V. Bapst, P. Kohli, M. Jaderberg, D. Hassabis, J. M. Jumper, *Nature* **2024**, *630* (8016), 493.

[10] M. Czjzek, S. Létoffé, C. Wandersman, M. Delepierre, A. Lecroisey, N. Izadi-Pruneyre, *J Mol Biol* **2007**, *365* (4), 1176.

[11] M. Bielecki, S. Antonyuk, R. W. Strange, J. W. Smalley, P. Mackiewicz, M. Śmiga, P. Stępień, M. Olczak, T. Olczak, *Biosci Rep* **2018**, *38* (5),

[12] J. L. Gao, A. H. Kwan, A. Yammine, X. Zhou, J. Trewhella, B. M. Hugrass, D. A. T. Collins, J. Horne, P. Ye, D. Harty, K. A. Nguyen, D. A. Gell, N. Hunter, *Nat Commun* **2018**, *9* (1), 4097.

[13] S. Zambolin, B. Clantin, M. Chami, S. Hoos, A. Haouz, V. Villeret, P. Delepelaire, *Nat Commun* **2016**, *7*, 11590.

[14] T. J. Bateman, M. Shah, T. P. Ho, H. E. Shin, C. Pan, G. Harris, J. E. Fegan, E. A. Islam, S. K. Ahn, Y. Hooda, S. D. Gray-Owen, W. Chen, T. F. Moraes, *Nat Commun* **2021**, *12* (1), 6270.

[15] R. Simon, U. Priefer, A. Puhler, *Bio/Technolgy* **1983**, *1* (9), 37.

[16] J. M. Ghigo, S. Létoffé, C. Wandersman, *J Bacteriol* **1997**, *179* (11), 3572.

[17] X. Wang, W. Liu, D. Zhu, L. Yang, M. Liu, S. Yin, M. Wang, R. Jia, S. Chen, K. Sun, A. Cheng, X. Chen, *BMC Genomics* **2014**, *15* (1), 479.

[18] L. Huang, M. Liu, D. Zhu, L. Xie, M. Huang, C. Xiang, F. Biville, R. Jia, S. Chen, X. Zhao, Q. Yang, Y. Wu, S. Zhang, J. Huang, X. Ou, S. Mao, Q. Gao, D. Sun, B. Tian, M. Wang, A. Cheng, *Front Microbiol* **2021**, *12*, 634895.

[19] C. Fournier, A. Smith, P. Delepelaire, *Mol Microbiol* **2011**, *80* (1), 133.
